# A Delphi study of current practices and establishing consensus regarding assessment of fitness to drive among patients with brain tumours

**DOI:** 10.1007/s11060-025-05030-z

**Published:** 2025-04-16

**Authors:** Adam H. Lapidus, Bianca Devitt, Harriet Herbison, Sophie Tran, Jen Cheung, Lucy Gately, Andrew Neal, Malaka Ameratunga

**Affiliations:** 1 Department of Oncology, Alfred Health, Melbourne, VIC Australia; 2 Department of Oncology, Eastern Health, Clinical School, Monash University, Melbourne, VIC Australia; 3 Centre for Health Economics, Monash Business School, Monash University, Melbourne, VIC Australia; 4 Department of Oncology, Western Health, Melbourne, VIC Australia; 5 School of Translational Medicine, Monash University, Melbourne, VIC Australia; 6 Personalised Oncology Division, Walter and Eliza Hall Institute, Melbourne, VIC Australia; 7 Department of Neuroscience, School of Translational Medicine, Monash University, Melbourne, VIC Australia

**Keywords:** Brain cancer, Driving, Motor vehicle accidents, Delphi, Guidelines

## Abstract

**Purpose:**

Evaluating fitness to drive among patients with brain tumours remains a challenge for clinicians. Due to difficulties in conducting prospective driving studies in this patient cohort, a Delphi study was performed to formulate new driving guidelines for patients with brain tumours.

**Methods:**

The survey questions, which were designed by utilising Australian driving guidelines and previous Delphi studies, established panelists’ expertise, and then used a 9-point Likert scale to formulate new driving guidelines. An expert group of panelists comprising medical oncologists, radiation oncologists, neurosurgeons, and neurologists were chosen based on membership to professional societies with validation in part one of the survey. Two rounds of anonymised surveys were performed using REDCap for data entry, and a novel automated methodology on R for data analysis.

**Results:**

46 statements regarding fitness to drive were developed. Among the 37 surveys distributed, there were 26 responses (70.3% response rate) from round one, and 17 responses (65.4% response rate) for round two. Among the 46 statements, 19 (41.3%) achieved consensus. In addition to establishing a framework for assessing patients, there was notable consensus agreement for stable imaging required as part of evaluation and the need for continual reassessment.

**Conclusion:**

Despite clinicians being aware of driving guidelines, determining fitness to drive among patients with brain tumours remains a challenge. This Delphi study identified consensus agreement for the need for stable imaging, and continually reassessing fitness to drive. These novel findings could be translated into future driving guidelines and consensus statements can be integrated into clinical practice.

**Supplementary Information:**

The online version contains supplementary material available at 10.1007/s11060-025-05030-z.

## Introduction

Driving is a complex, multisensory task, requiring intact neurocognitive skills and is crucial for autonomy and quality of life [1]. Patients with primary and metastatic brain tumours may have functional deficits from both the disease and treatment, which can impact their ability to drive [2]. Despite the legal, ethical, and medical responsibility of clinicians treating patients with brain tumours to determine fitness to drive, guidelines are heterogeneous and underpinned by limited evidence [3–5]. The Australian driving guidelines for assessing fitness to drive outline that patients with space-occupying lesions, including brain tumours, are considered unfit for an unconditional license if there is significant visuospatial, executive, cognitive, or motor impairment, whereas a conditional license may be considered following a treating doctor and practical driver assessment [3]. No specific recommendations are made based upon tumour histology, unlike United Kingdom (UK) guidelines [5]. While there are specific guidelines for epilepsy and visual deficits, as well as a general advice regarding time post intracranial surgery, guidelines do not provide clear guidance regarding treatment toxicity, tumour progression, or the utility of imaging as part of assessment [3].

Consequently, there can be an inconsistent approach to driving restriction in patients with brain tumours, which can be problematic given the inherent multi-disciplinarity of neuro-oncology, which can result in conflicting advice from different specialists. We recently conducted a systematic review evaluating the relationship of driving with brain tumours and incidence of motor vehicle crashes (MVC), identifying significant gaps in the literature regarding MVC risk in this patient population [6]. One of the primary challenges in formulating accurate driving guidelines is the lack of real-world data establishing the true risks posed by patients with brain tumours driving, as most research in this area concerns clinician attitudes [6].

Given the paucity of existing data, the difficulties in conducting prospective studies in this patient population, and potential gaps in the current driving recommendations, developing consensus recommendations needs to be achieved through alternative modalities such as the Delphi technique [7]. The Delphi technique is a structured, systematic process of attaining reliable consensus when research is limited or practically difficult [8]. The key elements of a Delphi methodology include selecting a group of expert panelists, maintaining anonymity of panelists to minimize bias and conformity, providing controlled feedback, and an iterative design whereby subsequent rounds are informed by the results of previous rounds [8]. A previous Delphi study, performed by the Swiss Neuro-Oncology Society (SwissNOS) and the Swiss Society for Legal Medicine (SGRM), identified criteria considered incompatible with driving and formulated structured guidelines for driving assessment [9]. The Swiss study identified non-compensated palsies, neglect, altered consciousness state, progressive disease on magnetic resonance imaging (MRI), visual anopsia, patient unreliability, epileptic-specific potentials on electroencephalogram (EEG) and neuropsychological changes as incompatible with driving [9]. However, there is no equivalent study for the Australian population.

Consequently, a two round Delphi study was performed using an expert group of medical oncologists, radiation oncologists, neurosurgeons, and neurologists, with the aim of gaining insight into current attitudes of treating clinicians and formulating new driving guidelines for clinicians to use as part of the assessment of fitness to drive in patients with brain tumours.

## Methodology

The questions for the Delphi survey were developed from the existing Australian guidelines, informed by our previous systematic review [6] and the previous modified Delphi Survey performed by the SwissNOS and SGRM [3, 9]. The statements were devised to firstly address the key elements of current Australian driving guidelines, and secondly to explore potential improvements previously identified by the Swiss Delphi survey. Questions were revised by a small group of neurologists and medical oncologists prior to being distributed to panelists, to ensure clinical relevance. Panelists were identified through professional networks and were only included if they had an active membership to a professional society and specialist qualification such as Royal Australasian college of Physicians (RACP) for medical oncologists and neurologists, Royal Australasian College of Surgeons (RACS) for neurosurgeons, and Royal Australian and New Zealand College of Radiologists (RANZCR) for radiation oncologists. The expertise of included specialists was validated in the round one to ensure panelists were actively treating at least one patient annually with brain tumours. There were no predefined criteria regarding minimum experience or volume of brain tumours seen annually.

For round one, the questions were divided into three sections. The first section established panelists’ expertise. The second section surveyed clinicians’ current patterns of care regarding driving restrictions. The third section used a 9-point Likert scale for the devised questions [7]. A systematic review on conducting and reporting Delphi Studies in a palliative care setting found that most studies had either two or three rounds (22/30), and most cut-offs for consensus were set as either 75% or 80% [7]. Consequently, statistically, the results were analyzed using an interquartile range (IQR), with consensus defined as 75% or more panelists selecting a score of three or lower (i.e. not important), or seven or higher (i.e. very important) for each statement. Statements that did not result in a consensus after round one were revised and modified according to panelist responses. Responses were anonymised.

Round one of the surveys was open from 13/08/2024 to 13/09/2024, while round two was open from 11/11/2024 to 19/01/2025. The survey was distributed using REDCap Helix [10, 11]. Data was exported from REDCap to RStudio program for analysis. Using an automated approach, individualised summary data was generated for each respondent including the results of all the questions and plots containing the IQR, median, lower and highest ratings, as well as the individual participant’s response to allow panelists to understand where their views ranked relative to the group consensus. Additionally, panelists were provided with a qualitative summary of the results of statements that did achieve consensus. An example of this summary data is included in Supplementary Appendix B.

Panelists who responded to round one were subsequently invited to round two of the Delphi study. Questions for round two were divided into two large subsections: questions from the previous survey that did not meet consensus, and new questions based on feedback and free text responses from the previous survey. Some of the questions from round one that were not close to consensus were reworded in response to the free text responses from round one. Data was analysed using RStudio program version 4.3.3. The research (project number 85/24) was approved by the Alfred Human Research Ethics Committee on 11 June 2024.

## Results

**Fig. 1:**
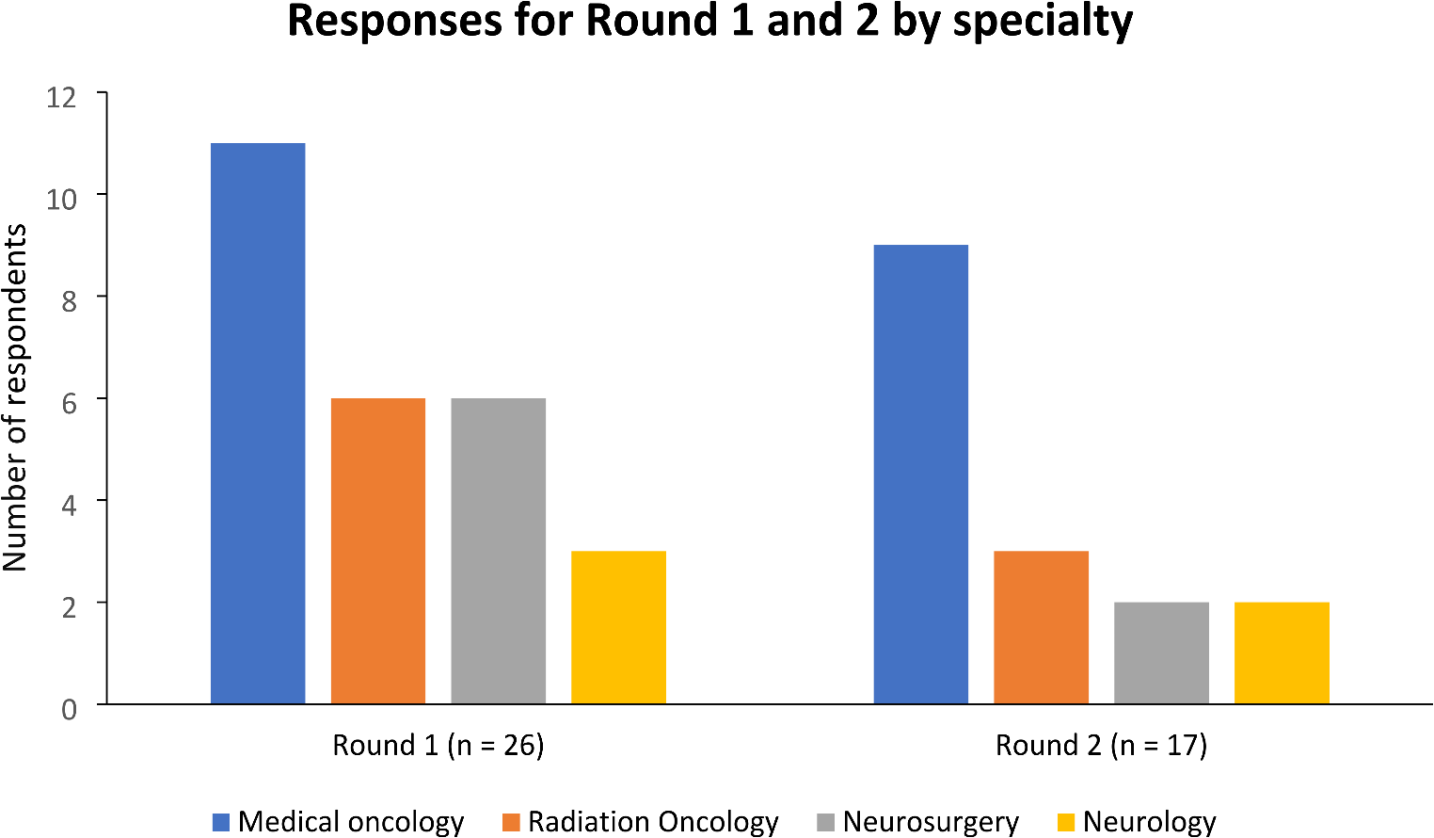
Breakdown of respondents by subspecialty in round one and round two

**Fig. 2:**
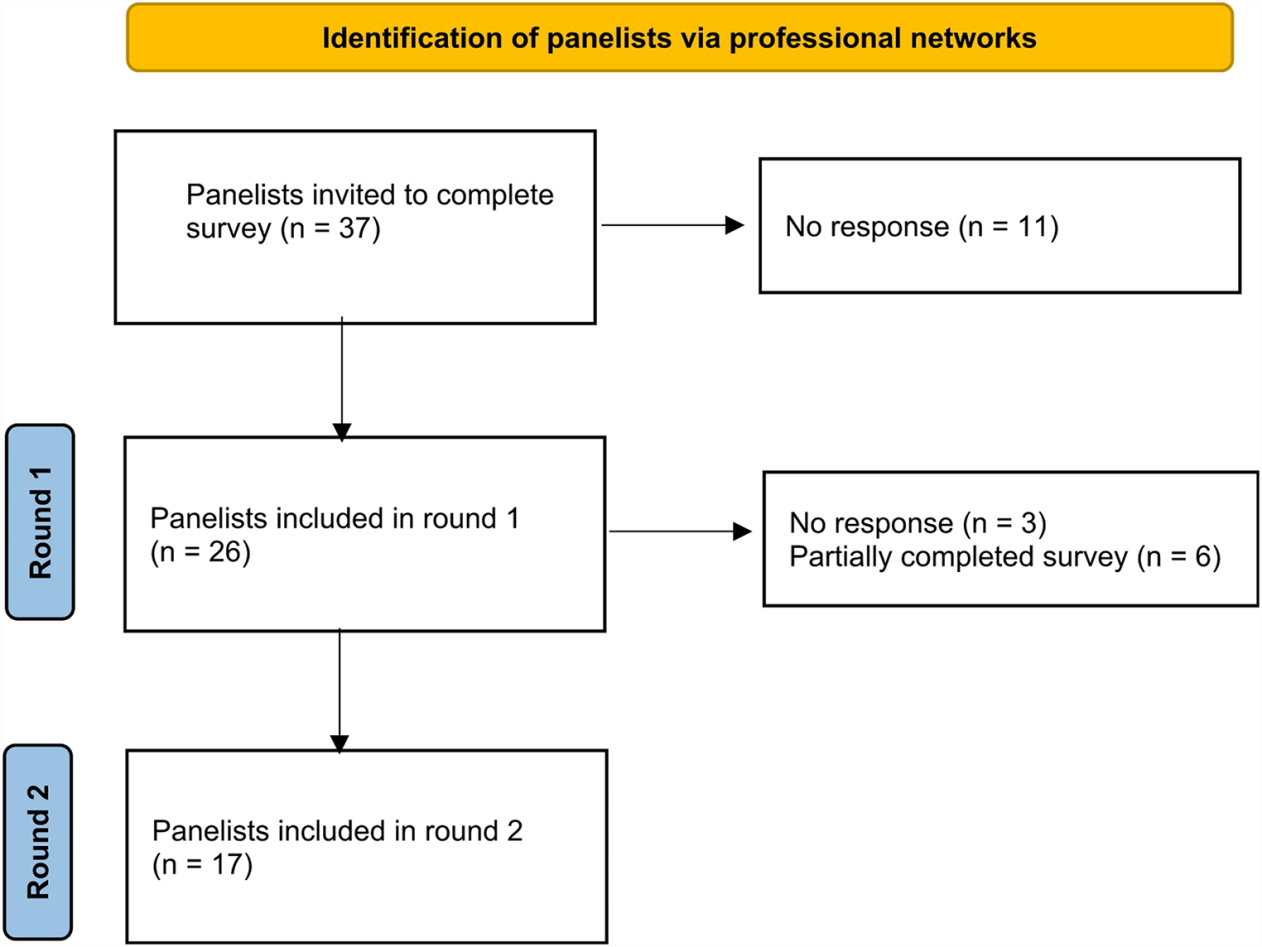
Flowchart summarizing Delphi survey rounds

37 surveys were distributed in round one, which yielded 26 responses (70.3% response rate), while round two yielded 17 responses (65.4% response rate). A bar graph displaying the breakdown of responses based on subspecialties for both rounds is displayed in Fig. 1. A flow chart of panelist inclusion in the Delphi study is shown in Fig. 2.

The demographics of the included panelists are reflected in Table 1. The panel was multidisciplinary, comprising 11 oncologists (42.3%), six radiation oncologists (23.1%), six neurosurgeons (23.1%), and three neurologists (11.5%). Most panelists (88.5%) worked in tertiary referral hospitals, which can be defined as a hospital providing a wide range of services including specialist units with large patient cohorts [12]. 23.1% of panelists had over 20 years’ experience, 19.2% had between 11 and 20 years’ experience, and 32.3% had between six to ten years’ experience. Among the nine panelists that did not respond to round two were two medical oncologists, two radiation oncologists, four neurosurgeons, and one neurologist. Among the panelists who did not respond for round 2, 77.8% saw over 30 patients with brain tumours annually, 77.8% had over ten driving queries annually, while 22.2% had over 20 years’ experience.

In terms of the panelists’ practices, as shown in Tables 1, 2 and 76.9% respondents reported treating over 30 patients with brain tumours per annum and 76.9% also had over ten driving queries from patients with brain tumours each year. Data was not collected on the main tumour types treated. When addressing driving restrictions among patients with brain tumours, 53.8% of respondents did so on an as-needed basis, whereas 46.2% discussed with every patient. Notably, there were differing practices in terms of specialties, whereby 36.4% of medical oncologists, 33.3% of radiation oncologists, 66.7% of neurosurgeons, and 66.7% of neurologists discussed driving restrictions with every patient. Nearly all panelists (96.2%) were aware of driving guidelines, yet 92.3% felt there needed to be more specific driving guidelines. 53.8% of respondents have had to impose driving restrictions on a patient that had prior clearance by a previous doctor. Specifically, 45.5% of medical oncologists, 66.7% of radiation oncologists, 50% of neurosurgeons, and 66.7% of neurologists had to override previous clearance. For most respondents, the potential for seizures was considered more influential in decision making compared to visual field, sensory, or motor deficits. The free text responses for the four panelists who answered ‘other’ were integrated into questions for round two of the survey, none of which achieved consensus. Among the free text responses were (1) the answers depend on the patient and that a driving assessment is only necessary if there is a neurological or cognitive deficit, (2) the need to demonstrate insight, and (3) asymptomatic brain metastases who remain at risk of acute neurological deficit. One of the respondents listed multiple potential factors including objective cognitive assessment, right foot drop if automatic transmission, and continual clinical and imaging surveillance. Additionally, the respondent advised to consideration of neck mobility as well as daytime versus nighttime driving, rural versus metropolitan driving.

**Table 1:** Respondent demographics (*n* = 26)

Questions	*N* (%)
**Age (years)**	
30–39	4 (15.4%)
40–49	13 (50.0%)
50–59	8 (30.8%)
60+	1 (3.8%)
**Main practice site**	
Tertiary referral hospital Regional hospital Private hospital	23 (88.5%) 2 (7.7%) 1 (3.8%)
**Specialty**	
Medical oncology Radiation oncology Neurosurgery Neurology	11 (42.3%) 6 (23.1%) 6 (23.1%) 3 (11.5%)
**Years practicing as a specialist (years)**	
0–5 6–10 11–20 > 2	4 (15.4%) 11 (42.3%) 5 (19.2%) 6 (23.1%)
**Number of patients with brain tumours seen per annum**	
1–10 11–20 21–30 > 30	2 (7.7%) 3 (11.5%) 1 (3.8%) 20 (76.9%)

**Table 2:** Respondent approaches to driving restrictions among patients with brain tumours (*n* = 26)

*Questions*	*N* (%)
**Frequency of driving queries received from patients with brain tumours per annum**	
1–10	6 (23.1%)
> 10	20 (76.9%)
**Difficulty answering queries relating to driving restrictions**	
Yes No	14 (53.8%) 12 (46.2%)
**Aware of driving guidelines**	
Yes No	25 (96.2%) 1 (3.8%)
**There needs to be more specific driving guidelines**	
Yes No	24 (92.3%) 2 (7.7%)
**Address driving restrictions with every patient that has a brain tumour or on an as-needed basis**	
Every patient As-needed basis	12 (46.2%) 14 (53.8%)
**Response in cases of non-compliance with driving restriction**	
Contact the patient directly Contact a licensing authority Contact both the patient and the licensing authority No action taken	13 (50.0%) 0 (0.0%) 13 (50.0%) 0 (0.0%)
**Primary symptom/sign influencing recommendation to restrict/permit driving**	
Potential for seizures Visual field deficit Sensory deficit Motor deficit Other	16 (61.5%) 6 (23.1%) 0 (0.0%) 0 (0.0%) 4 (15.4%)
**Ever needed to impose restrictions on a patient despite patient receiving approval previously from a different doctor**	
Yes No	14 (53.8%) 12 (46.2%)

Overall, 19 statements achieved consensus: 15 statements had consensus agreement, and four had consensus disagreement. The statements which achieved consensus for round one and two are displayed in Table 3. Conversely, 27 unique statements did not achieve consensus over round one and two, and are displayed in Appendix A.

After two rounds of surveys, the consensus items could be grouped into (1) patient characteristics, (2) tumour characteristics, (3) change in disease status, and (4) clinical assessment. Of the 19 items, 12 align with the current Australian driving guidelines. The additional seven items identified by this study are new recommendations. Conversely, among statements which did not achieve consensus, seven statements addressed features present in the current guidelines. These statements primarily concerned (1) patient characteristics, and (2) perspectives on current epilepsy guidelines.

**Table 3:** Questions that achieved consensus and if the consensus aligns with Australian guidelines

Question	Consensus type (agree or disagree)	Consensus aligns with current guidelines (yes or no)
**Patient characteristics**		
For determining fitness to drive: A defined seizure free period is necessary.	Agree	Yes
The presence of ongoing seizures is incompatible with driving.	Agree	Yes*
The presence of hemianopia is incompatible with driving.	Agree	Yes
Reduced mini − mental state examination (MMSE) with a score of 10 − 20 is incompatible with driving.	Agree	No
Reduced MMSE with a score of < 10 is incompatible with driving.	Agree	No
Impaired visuospatial function is incompatible with driving.	Agree	Yes**
A defined period post − operatively if relevant is necessary.	Agree	Yes
Impaired executive function is incompatible with driving.	Agree	Yes
The use of anti − epileptic medications is incompatible with driving.	Disagree	Yes
**Tumour characteristics**		
Glioblastoma tumour histology is incompatible with driving.	Disagree	Yes
Metastatic carcinoma histology is incompatible with driving.	Disagree	Yes
**Change in disease status**		
For determining fitness to drive: A stable repeat CT brain or MRI brain is necessary.	Agree	No
The reduction or cessation of antiseizure medications should significantly influence driving restrictions for patients with brain tumours.	Agree	Yes
**Clinician assessment**		
For determining fitness to drive: A comprehensive neurological history is necessary.	Agree	No
For determining fitness to drive: A neurological examination is necessary.	Agree	No
Baseline (at the time of the initial driving assessment) visual fields are necessary.	Agree	Yes
I think it is important to continually reassess fitness to drive.	Agree	No
I believe the reassessment should follow the same principles as the original assessment.	Agree	No
EEGs should be considered mandatory as part of the assessment to determine fitness for driving among brain cancer patients.	Disagree	Yes

* Generally, patients are not permitted to drive if ongoing seizures, however guidelines may permit driving if ‘safe’ seizures

** Guidelines specify significant visuospatial deficits are prohibited

## Discussion

Whilst there have been multiple previous surveys which have assessed clinician opinions on driving regulations in brain cancer patients [13–16], including two surveys in Australian populations [17, 18], this is the first Delphi study to both assess clinician attitudes and then identify areas for change in driving guidelines. Moreover, the multidisciplinary nature of this study is unique, as previous studies [9, 19] did not include radiation oncologists or neurosurgeons as part of analysis. Among the strengths of this study are the proven credentials of the expert panel, as evidenced by the numbers of years practicing as a specialist, number of patients with brain tumours reviewed each year, and frequency of driving inquiries from patients with brain tumours each year. The expertise of the panelists in our study is highlighted by the near universal awareness of driving guidelines (96.2%), compared with 26.1% and 76% of panelists used in previous Australian surveys (16,17). Yet, despite this, over half advised difficulty answering driving restriction queries and only raised restrictions on an as-needed basis. Furthermore, nearly all believed there needed to be more specific driving guidelines. Interestingly, eight (72.7%) medical oncologists had difficulty answering queries relating to driving restrictions, whereas only three (50%) radiation oncologists, two neurosurgeons (33.3%), and one neurologist (33.3%) reported as such. These differences could be explained by the existence of separate guidelines for seizures and post-operative restrictions. Unfortunately, as suggested in our previous systematic review [6], there is limited evidence underpinning driving guidelines, and insufficient understanding of the risks in brain tumour patients driving.

There were several novel findings from this Delphi survey. Firstly, despite the lack of framework within current driving guidelines for clinicians when initially determining fitness to drive in patients with brain tumours, there was consensus among clinicians that taking a comprehensive neurological history, performing a neurological exam, and documenting baseline visual fields should form the basis of the assessment. Although taking a thorough neurological history and reassessing patients may be implied in the guidelines, they are not a requirement. The specific components of the neurological exam and visual fields were not assessed as part of the study, however possible exam findings were evaluated, as reflected in Table 3 and Appendix A. Beyond not mandating EEGs as part of the assessment, the study also affirmed the guideline’s stance on having a defined post-operative period of no driving; however current guidelines are advisory only, allowing neurosurgeons to have discretion with deciding duration. While there was disagreement regarding whether each tumour subtype needed its own specific guideline, there was consensus that specific tumour histology such as glioblastoma and brain metastases, did not prohibit driving [3]. However, in contrast to existing Australian guidelines, there was consensus agreement among panelists that stability on MRI or CT should be part of the assessment in patients with brain tumours. Stability on brain imaging was not defined as part of this study, however this should be based on the Response Assessment in Neuro-Oncology (RANO) criteria in which stable disease defined as clinically stable without evidence of progression or complete or partial response, and stable non-enhancing lesions, on the same or reduced dose of corticosteroids [20, 21]. RANO criteria do not advise fixed intervals for repeating scans but rather recommend an individualized approach based on the clinical scenario. The frequency of repeating scans was not assessed as part of this study and remains a complex decision requiring clinical discretion based on the patient’s symptoms, and future studies are needed to determine the optimal interval in cases where patients remain asymptomatic. Additionally, there was consensus that it is important to continually reassess fitness to drive, following the same principles as the original assessment. These are in line with the findings from the Swiss Delphi study reported in 2021, which suggested routine brain MRI, thorough history, and neurological examination, as well as neuropsychological and visual assessment when determining fitness to drive [9]. However, a more recent study by Mondia et al. formulated a wider multidisciplinary approach involving epileptologists, ophthalmologists, neuropsychologists and driving simulators to determining fitness to drive in brain cancer patients [19]. As part of their findings, Mondia et al. generated a decision tree which included some aspects of the current driving guidelines such as seizures, visual acuity impairment, visual field loss, diplopia, motor weakness, cognitive deficit, progressive disease, or concerns raised by patients or their families [19]. Nevertheless, these findings across studies highlight the wide range of clinically significant findings in determining fitness to drive, and the challenge of formulating a single driving guideline. There is important role for an Australian registry evaluating real world data, based on a previous Finnish study by Huuskonen et al., to quantify the true risks posed by patients with brain tumours [22]. Ultimately, further research into the true risks posed by patients with brain tumours driving, through a multidisciplinary lens, involving medical oncologists, radiation oncologists, neurologists, neurosurgeons, well as ophthalmologists, and occupational therapists is needed to resolve disagreements between guideline requirement and the expertise clinicians.

Secondly, this study highlights clinicians’ perspectives on aspects that are incompatible with driving. While several aspects align with the existing guidelines in terms of restricting patients with significant impairments in visuospatial perception, vision, memory, or executive function [3], consensus was not achieved regarding impairments in motor domains, sensory domains, or insight. Additionally, consensus was not achieved regarding current epilepsy guidelines for seizure-free period in brain tumours and following intracranial surgery. This suggests that existing guidelines may be too broad, and more granular information regarding deficits expected to impact the ability to drive safely should be incorporated into future guidelines. The study identified 19 statements which reached consensus, of which 12 align with existing Australian guidelines. The remaining seven statements signify opportunities to enhance clinical practice through future formal guideline revisions. Current guidelines do not entirely reflect the attitudes of practicing clinicians and further research is needed to resolve these disagreements with the current guidelines. Future studies at the local institutional level are necessary to validate these novel clinical assessment findings before they can be integrated into formal guidelines.

Thirdly, a striking finding from this Delphi survey was that over half of clinicians reported needing to impose restrictions on a patient, despite the patient previously receiving approval from a different doctor. All four specialties surveyed had between 45.5% and 66.7% of respondents needing to override previous clearance. Additionally, in cases of non-compliance, there is uncertainty around the most appropriate reporting with 50% contacting only the patient directly, and 50% also contacting a licensing authority. While this study focused on clinician attitudes towards assessing fitness to drive, patient perspectives were not specifically sought. A previous self-reported patient questionnaire by Mansur et al. identified that 88.4% of patients with brain tumours rated their driving as “good” or “excellent”, with only approximately 17.9% self-limiting their driving since their brain cancer with only one individual reporting concerns regarding their driving ability [23]. Beyond highlighting the possible directions for change in future driving guidelines, these findings amplify the challenges faced by clinicians who treat patients with brain tumours in balancing patient autonomy and confidentiality against clinical concern for their safety when driving. Future studies incorporating patient perspectives regarding driving restrictions or quality-of-life assessments would be valuable in ensuring patient-centered care when making these challenging decisions.

Finally, beyond the findings of the Delphi survey regarding assessing fitness to drive, the novel methodology of this study provides an innovative change for future Delphi studies. A laborious and time-intensive aspect of performing Delphi studies is analyzing, summarizing, and distributing the results between rounds in a timely manner [8]. R was a powerful tool in this study that accelerated the process of generating accurate results that are unique to each respondent. This study developed an automated methodology on R for inter-round analysis, that performed rapid data assessment, figure generation, and qualitive analysis. While previous Delphi studies have utilized R in scenario analysis, it does not appear that other Delphi studies have utilized R for the inter-round analysis [24]. This novel change to Delphi Methodology can be applied to future Delphi studies to more efficiently disseminate individualized inter-round summary results.

There are limitations to our Delphi survey. The total number of panelists that responded to both rounds is small (17), but nevertheless falls within the acceptable range for Delphi studies of eight to 20 panelists [25]. All the panelists work in Australia, and there appeared to be geographic selection bias as 88.5% respondents worked primarily in tertiary hospitals, with only 7.7% working in regional hospitals, which may limit the generalizability of the findings to rural populations. Yet current guidelines similarly do not distinguish between metropolitan and rural practice sites. There was an attrition of respondents between rounds one and two, with nine panelists not participating in round two. Although the experience level of non-responders was comparable to those who completed both rounds, 66.7% of neurosurgeons did not respond, which may have influenced the findings of round two. As most (27) statements did not achieve a consensus, the statements which did have consensus provide mostly a direction for possible improvements to guidelines. Unfortunately, the guidelines generated are broad and remain open to interpretation. Further studies are needed to refine the consensus statements, such as the frequency of follow up imaging required to establish stability. There may be a role for future multidisciplinary Delphi studies incorporating occupational therapists, to establish degrees of impairment. However, the strengths of our study include a multidisciplinary cross-section of clinicians involved in driving decision making and reasonably high response rates across both rounds at 70.3% for round one and 65.4% for round two.

## Conclusion

Determining fitness to drive among patients with brain tumours remains a challenge for medical oncologists, radiation oncologists, neurosurgeons, and neurologists. Despite the awareness of driving guidelines, there remains a demand for more specific guidelines. This Delphi Study identified 19 statements achieving consensus among panelists across four broad areas: (1) patient characteristics, (2) tumour characteristics, (3), change in disease status, and (4) clinician assessment. Pertinent novel findings include the need for stable repeat CT or MRI and the need to continually reassess fitness to drive following the same principles as the initial assessment. These findings can help inform future driving guidelines in Australia. The innovative automated methodology using R software demonstrated efficient inter-round quantitative and qualitative analysis and can be applied to future Delphi studies. A driving registry capturing real-world data may be required for quantifying the actual risks posed by patients with brain tumours. There remains uncertainty in instances of patient non-adherence and further research into the true risks of patients with brain tumours driving is needed to resolve disagreements between practicing expert clinicians and the current guidelines, especially in areas that did not achieve consensus.

## Electronic supplementary material

Below is the link to the electronic supplementary material.

Supplementary Material 1

Supplementary Material 2

## Data Availability

The datasets generated and/or analyzed during the current study are available from the corresponding author on reasonable request.
