# Supplementary material for: A Delphi study of current practices and establishing consensus regarding assessment of fitness to drive among patients with brain tumours
Source: J Neurooncol. 2025 Apr 16;173(3):645–53. doi: 10.1007/s11060-025-05030-z (PMC12170731; doi:10.1007/s11060-025-05030-z)
Supplement: Supplementary file 1 — Supplementary Material 1 [file 11060_2025_5030_MOESM1_ESM.docx]

**Supplementary material:**

Appendix A: Questions that did not achieve consensus and whether the questions are in Australian guidelines

| **Question** | **Criteria part of current guidelines (yes, no)** |
| --- | --- |
| **Patient characteristics** | |
| Neurological | |
| The presence of sensory neurological deficits is incompatible with driving. | Yes |
| A driving assessment is only necessary if there is a neurological or cognitive deficit. | No |
| Motor | |
| The presence of motor neurological deficits is incompatible with driving. | Yes |
| Among patients with brain tumours, the presence of lower limb or upper limb motor neurological deficits that interfere with the ability to drive is incompatible with driving. | Yes |
| The presence of right drop foot is incompatible with driving an automatic transmission car. | No |
| Cognitive | |
| The presence of cognitive neurological deficits is incompatible with driving. | No |
| Reduced speed of information processing is incompatible with driving. | No |
| Reduced MMSE with a score of 25 − 29 is incompatible with driving. | No |
| The unreliability of a patient is incompatible with driving. | No |
| For determining fitness to drive the patient must have intact cognitive function. | No |
| The inability of patients to demonstrate insight is incompatible with driving. | Yes |
| Epilepsy/seizure | |
| The presence of an EEG with epilepsy − specific potential is incompatible with driving. | No |
| Visual | |
| The presence of superior quadrantanopia is incompatible with driving. | Yes |
| The presence of inferior quadrantanopia is incompatible with driving. | Yes |
| **Tumour characteristics** | |
| Each brain tumour subtype needs its own specific recommendation. | No |
| Patients with multiple brain metastases require different guidelines to patients with single primary brain tumours. | No |
| The WHO grade of glioma is an important determinant when determining fitness to drive. | No |
| **Change in disease status** | |
| The status of active treatment or the time since the last treatment should significantly impact driving restrictions for patients with brain tumours. | No |
| Patients with asymptomatic brain metastases who remain at risk of acute neurology deficits should be more frequently reassessed for driving fitness. | No |
| **Clinician assessment** | |
| For determining fitness to drive: A formal visual assessment is necessary. | No |
| For determining fitness to drive: A baseline (at the time of initial driving assessment) neurological assessment with a focus on safe driving is necessary. | No |
| For determining fitness to drive: A driving assessment with a driving instructor / occupational therapist is necessary. | No |
| Patients should sign a document with the recommendation of their physician. | No |
| Patients who are advised not to drive by their physician should sign a document that will be sent to the appropriate driving authority. | No |
| For determining fitness to drive, a neurological examination that is tailored to the known location of the tumour is necessary. | No |
| MMSE is not a useful tool for determining fitness to drive among patients with brain cancer. | No |
| **Perspectives on current guidelines** | |
| The current epilepsy guidelines for seizure − free period are adequate for patients with brain tumours / following intracranial surgery. | Yes |

Appendix B: Example of summary data from round 1 of the Delphi Survey generated on R
