# Supplementary material for: A Delphi study of current practices and establishing consensus regarding assessment of fitness to drive among patients with brain tumours
Source: J Neurooncol. 2025 Apr 16;173(3):645–53. doi: 10.1007/s11060-025-05030-z (PMC12170731; doi:10.1007/s11060-025-05030-z)
Supplement: Supplementary file 2 — Supplementary Material 2 [file 11060_2025_5030_MOESM2_ESM.pdf]

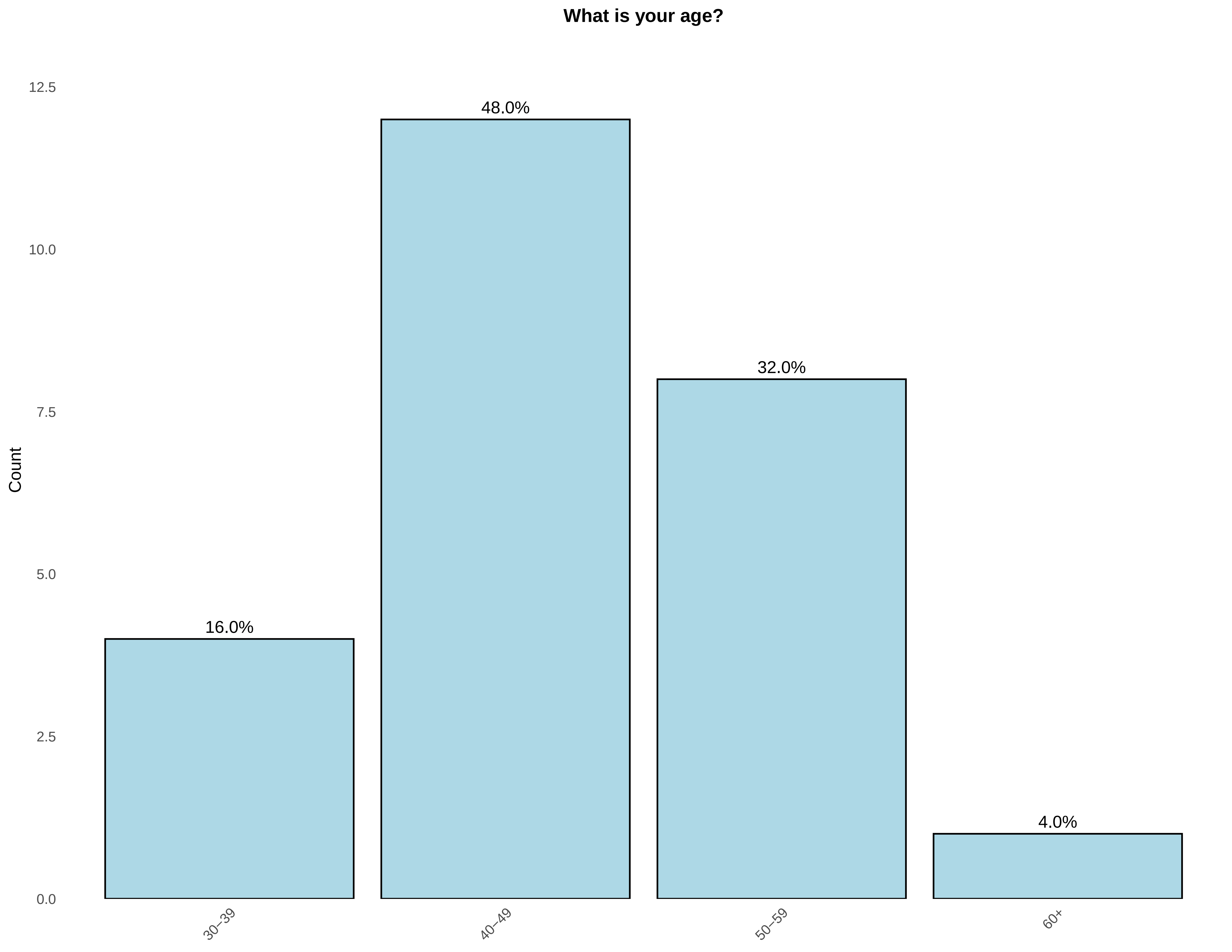
What is your age?
Count
12.5
10.0
7.5
5.0
2.5
0.0
48.0%
32.0%
16.0%
4.0%
30–39
40–49
50–59
60+

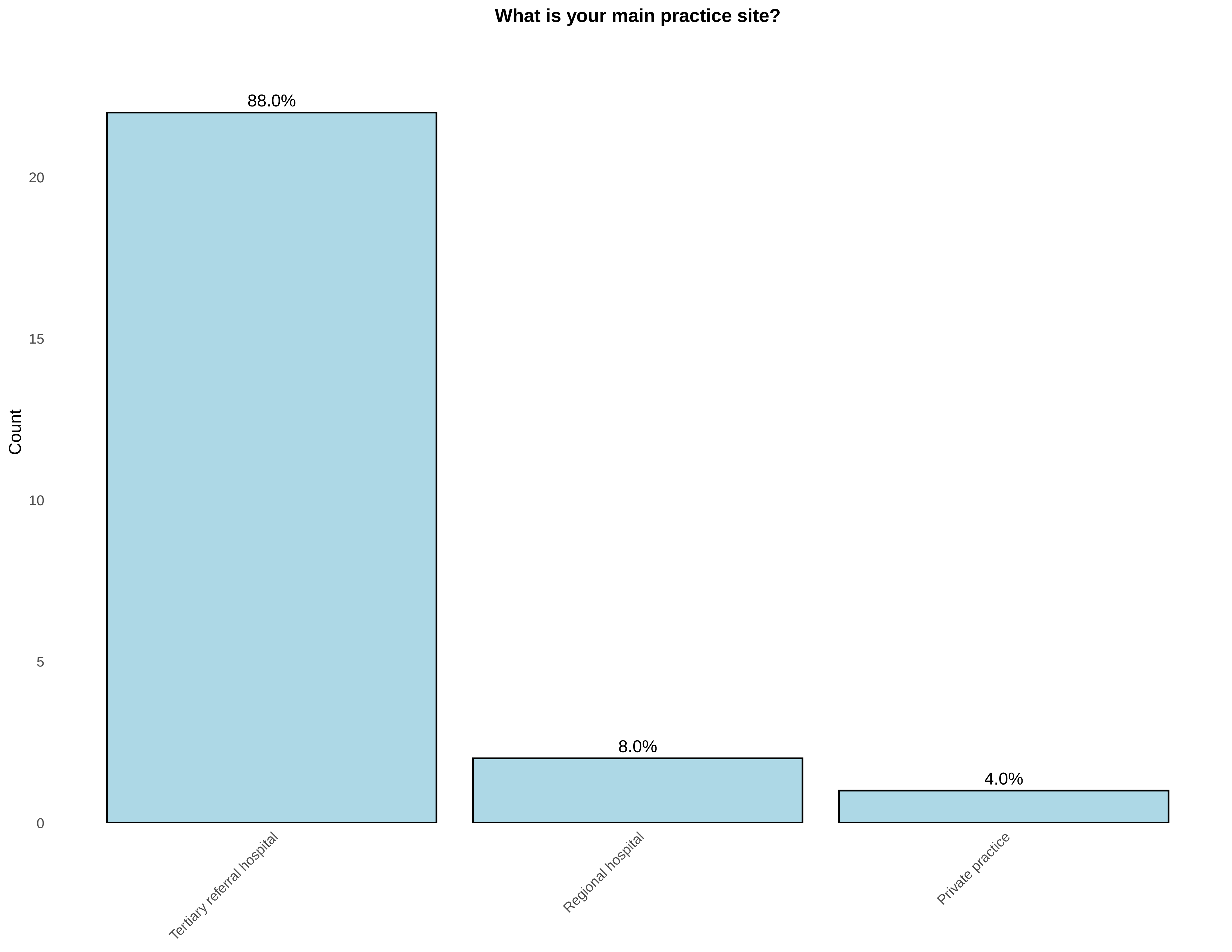
What is your main practice site?
88.0%
8.0%
4.0%
Count
0
5
10
15
20
Tertiary referral hospital
Regional hospital
Private practice

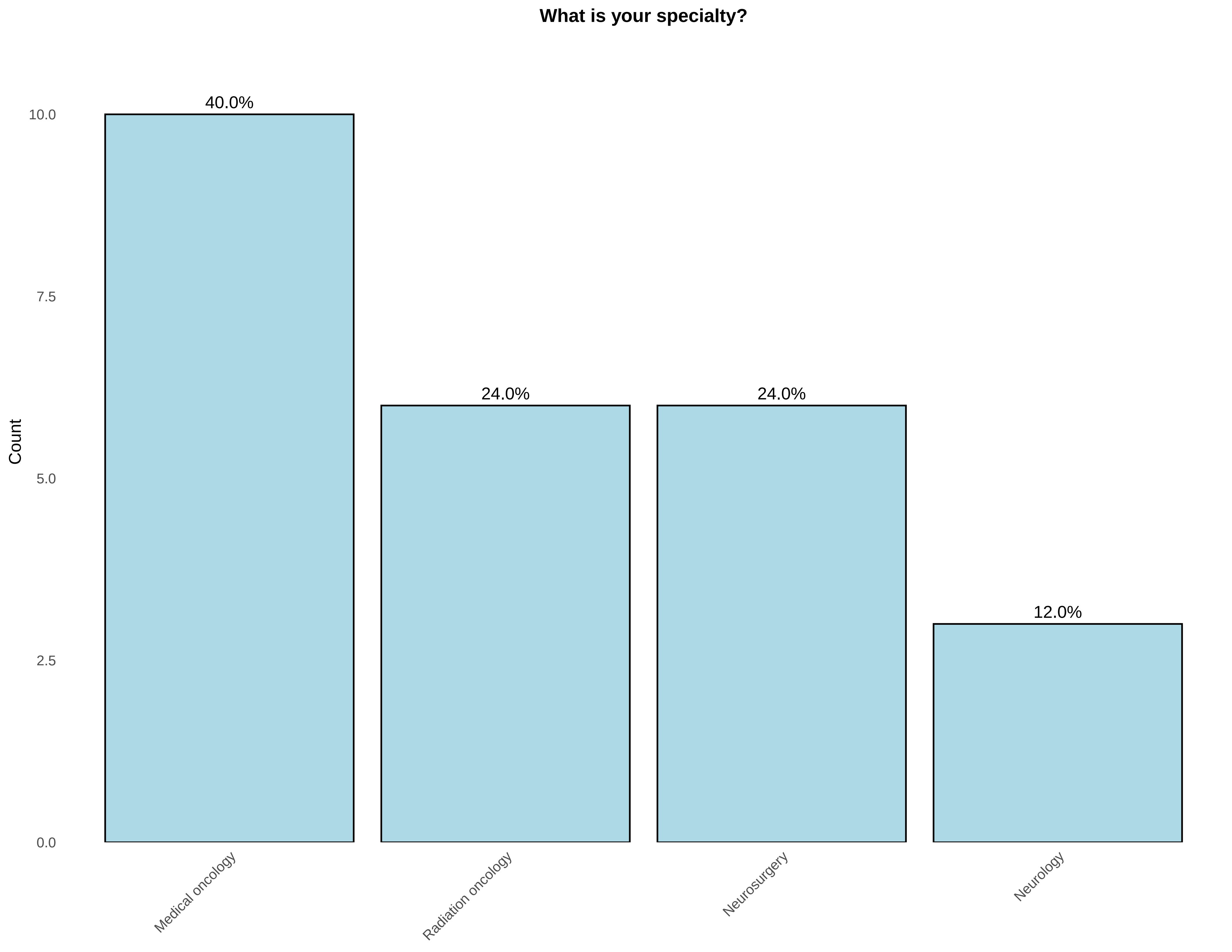
What is your specialty?
Count
10.0
7.5
5.0
2.5
0.0
40.0%
24.0%
24.0%
12.0%
Medical oncology
Radiation oncology
Neurosurgery
Neurology

How many years have you been practicing as a specialist?

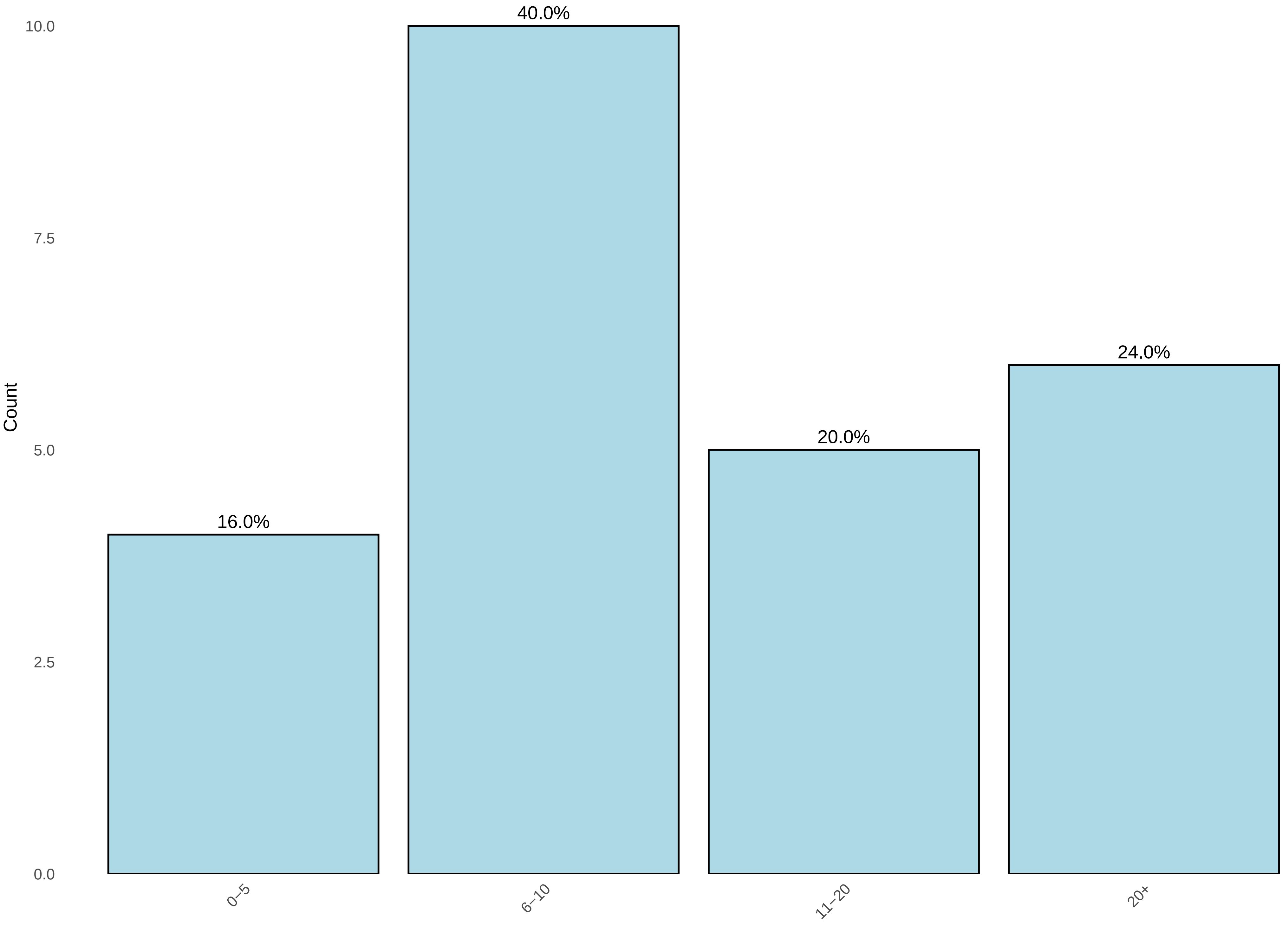

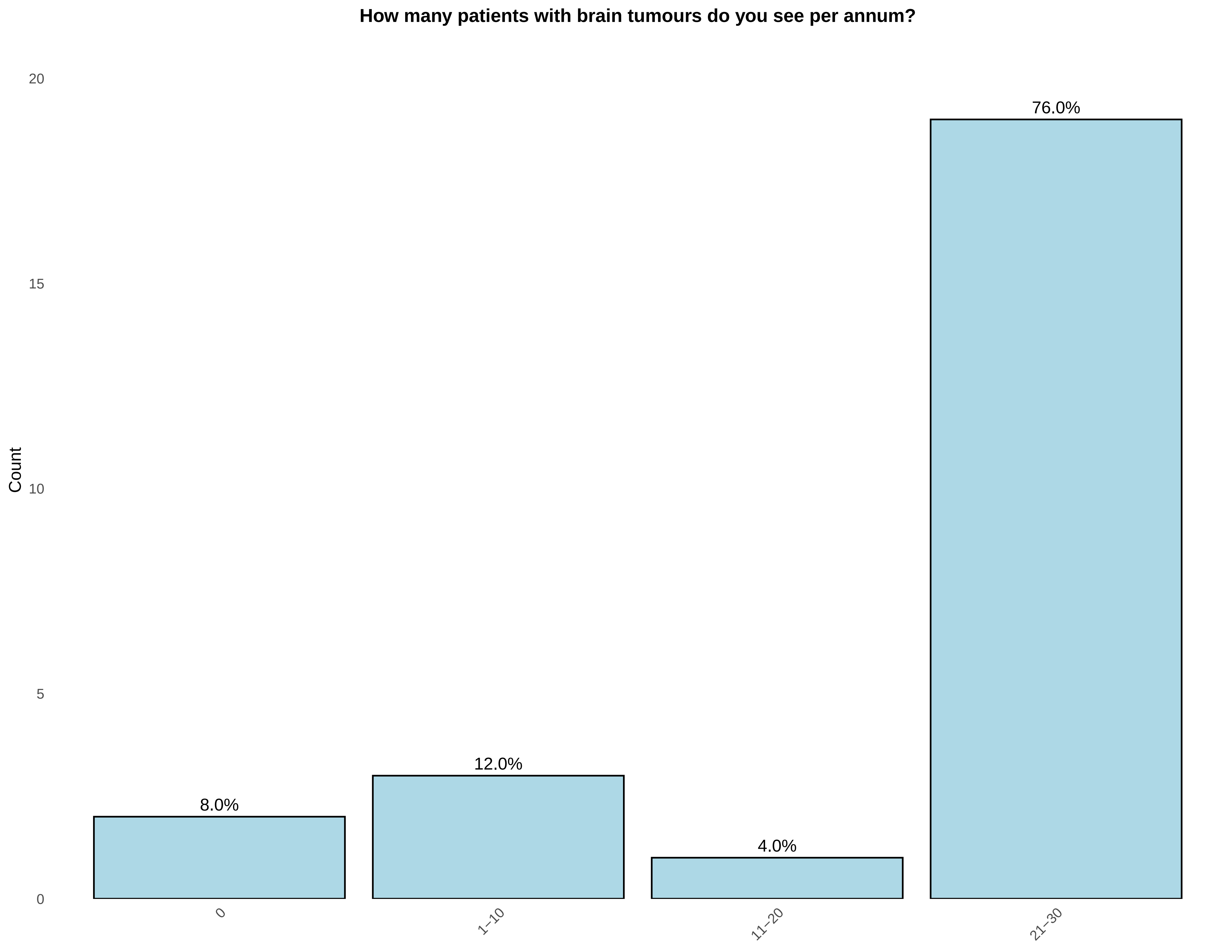
How many patients with brain tumours do you see per annum?
Count
20
15
10
5
0
76.0%
12.0%
8.0%
4.0%
0
1–10
11–20
21–30

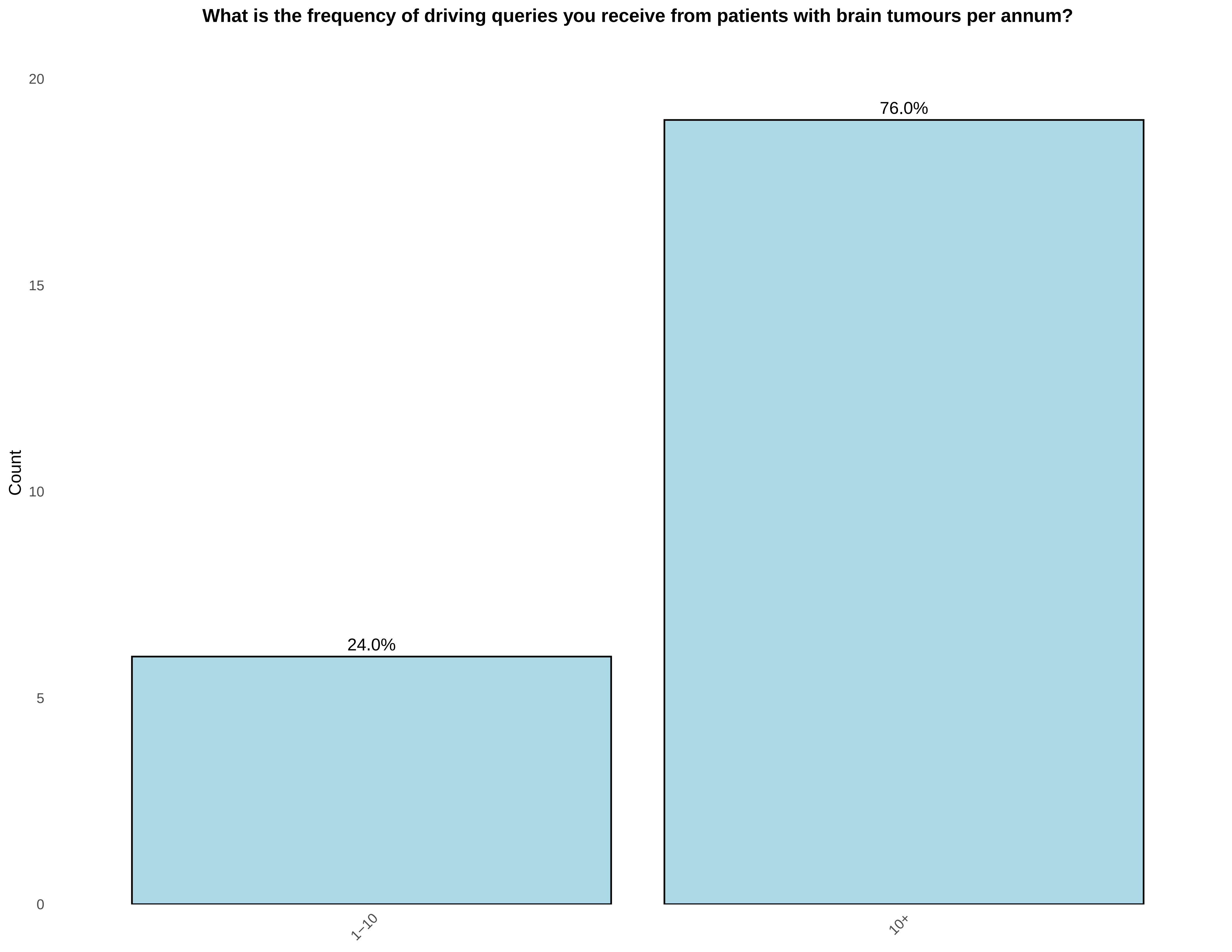
What is the frequency of driving queries you receive from patients with brain tumours per annum?
Count
20
15
10
5
0
76.0%
24.0%
1–10
10+

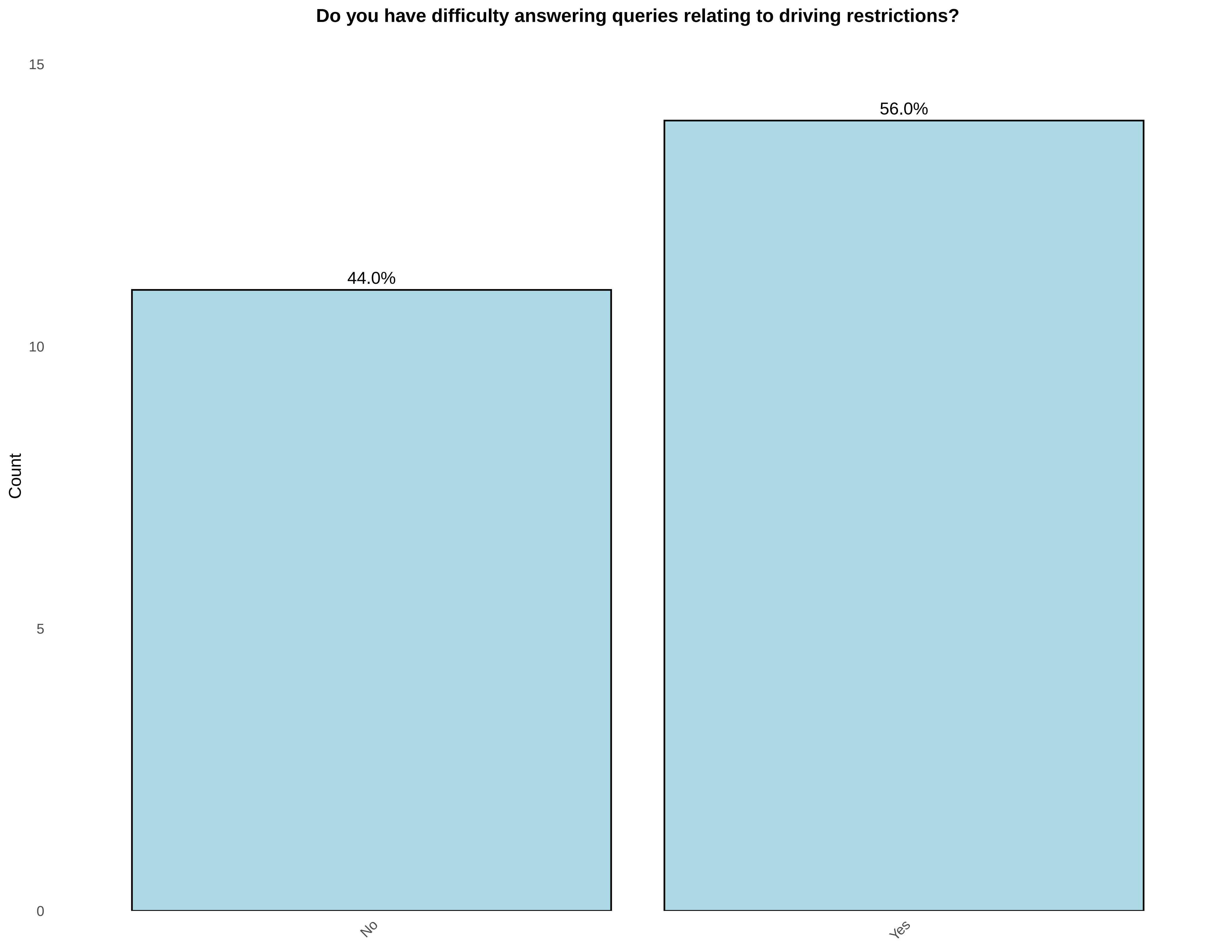
Do you have difficulty answering queries relating to driving restrictions?
Count
15
10
5
0
44.0%
56.0%
No
Yes

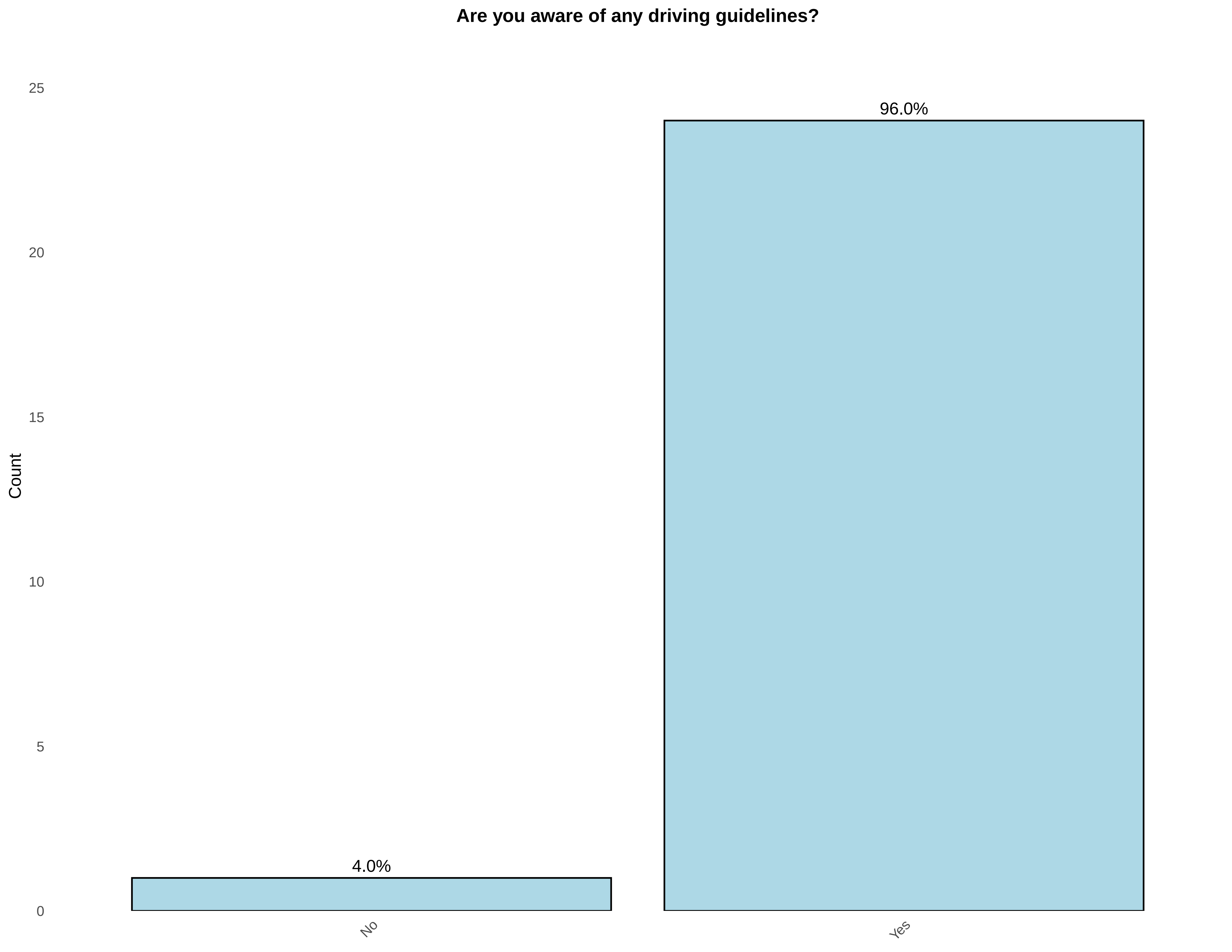
Are you aware of any driving guidelines?
Count
25
20
15
10
5
0
96.0%
4.0%
No
Yes

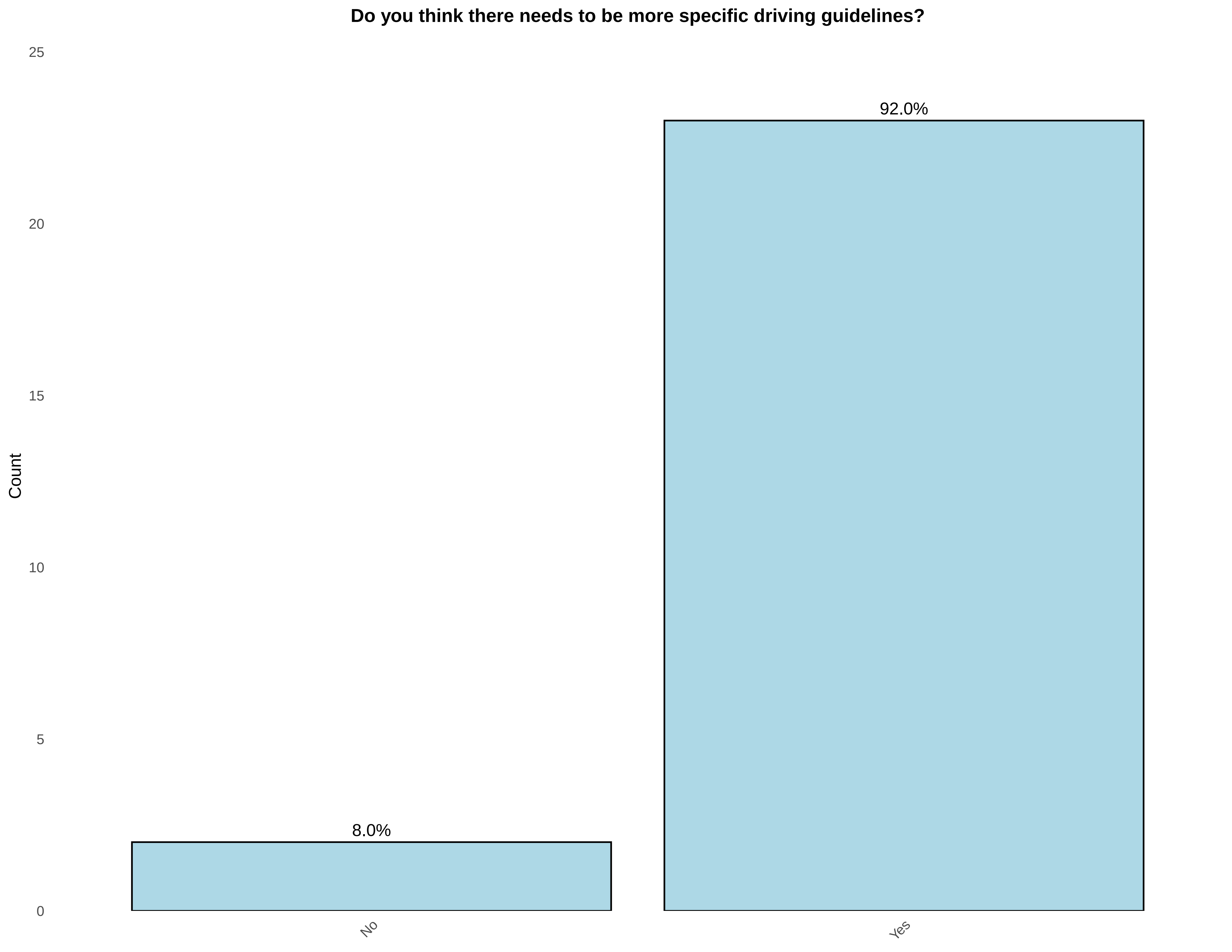
Do you think there needs to be more specific driving guidelines?
Count
25
20
15
10
5
0
92.0%
8.0%
No
Yes

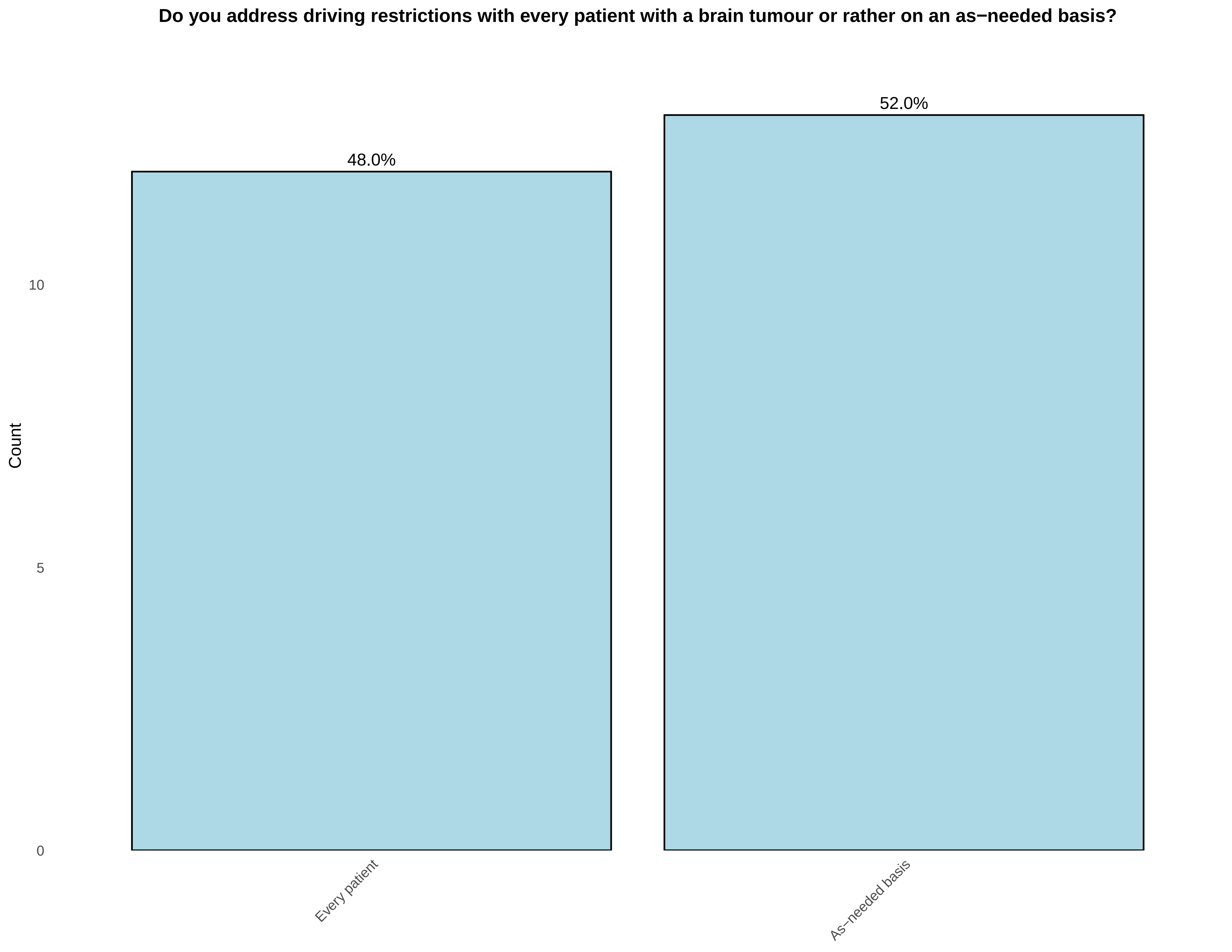
Do you address driving restrictions with every patient with a brain tumour or rather on an as−needed basis?
Count
0
5
10
48.0%
52.0%
Every patient
As−needed basis

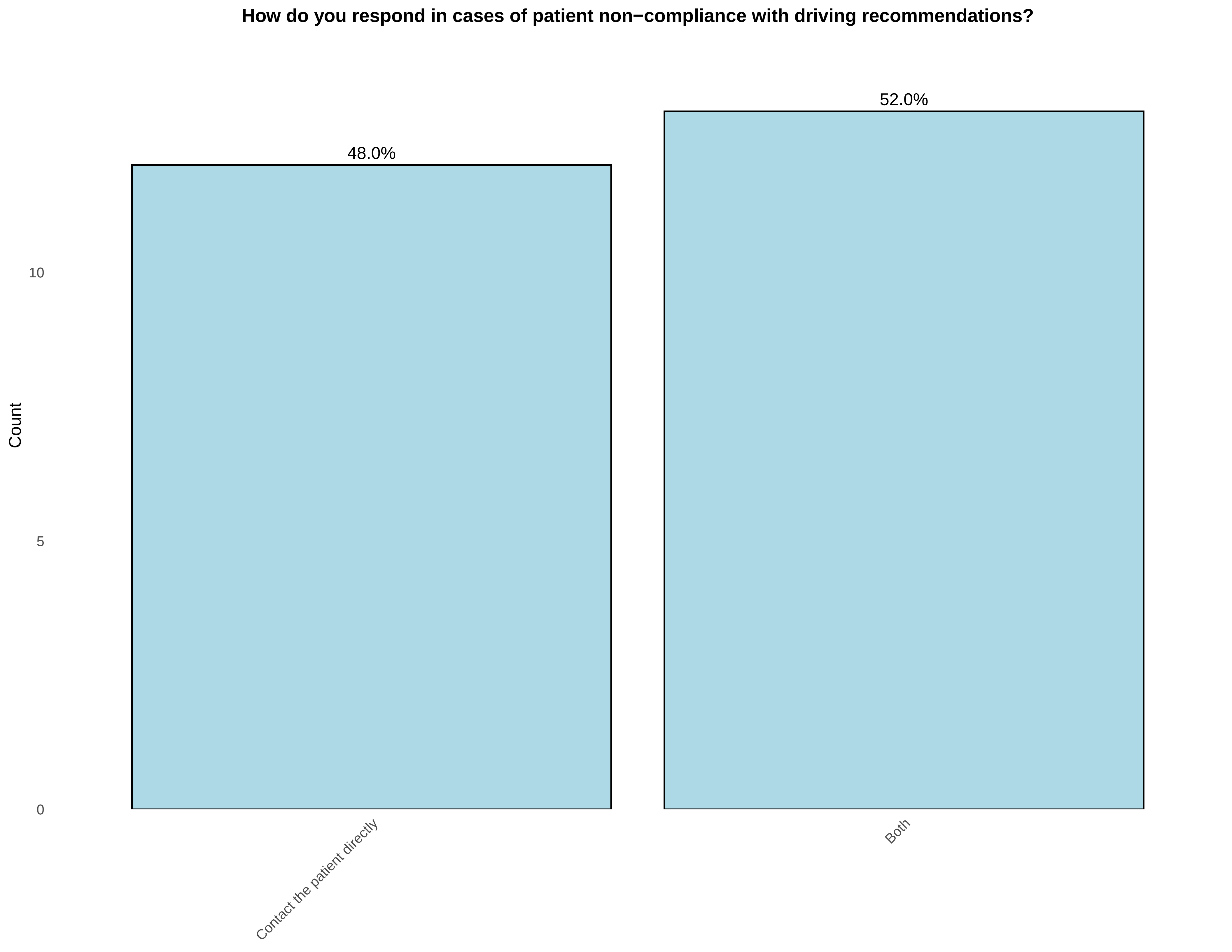
How do you respond in cases of patient non−compliance with driving recommendations?
52.0%
48.0%
Count
10
5
0
Contact the patient directly
Both

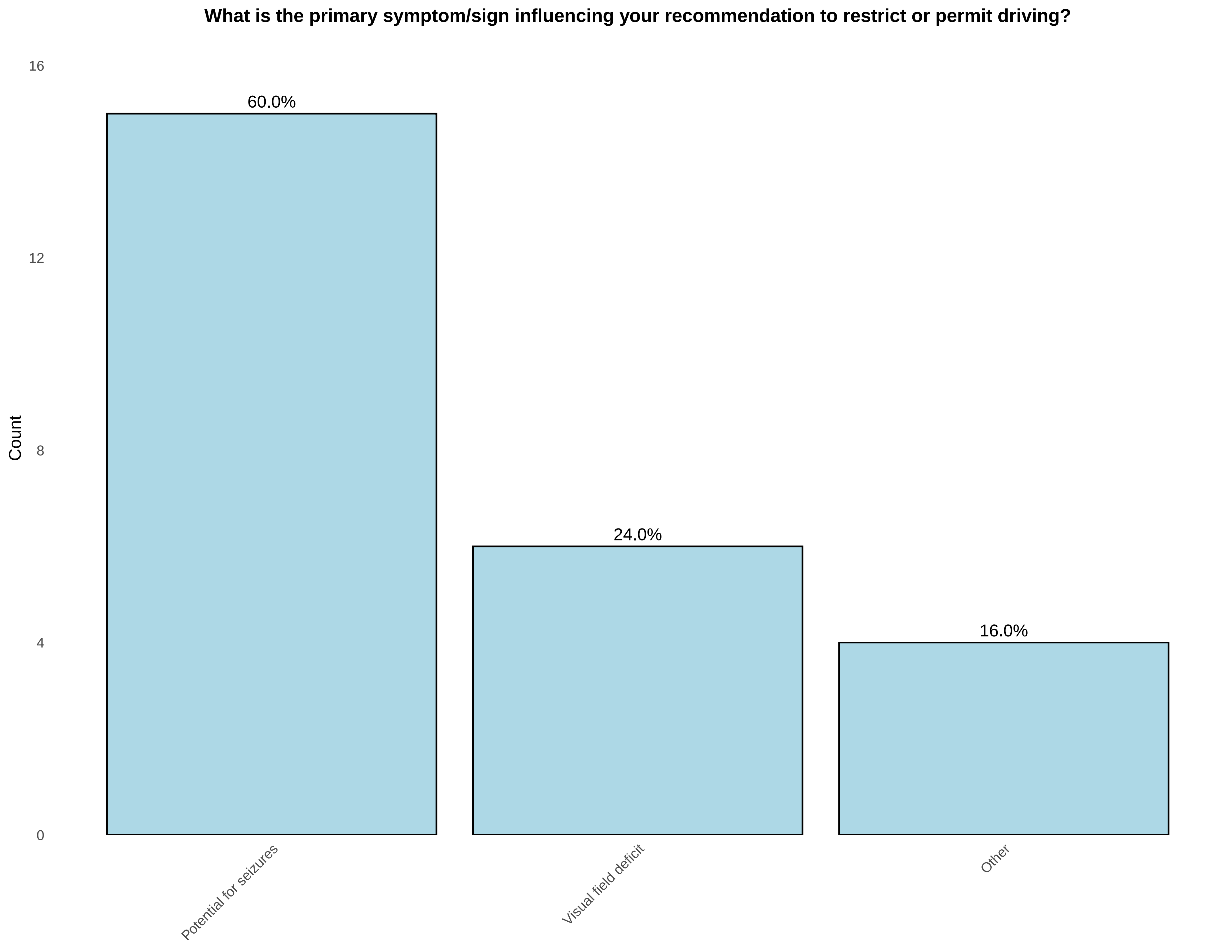
What is the primary symptom/sign influencing your recommendation to restrict or permit driving?
Count
16
12
8
4
0
60.0%
24.0%
16.0%
Potential for seizures
Visual field deficit
Other

Have you ever had to impose driving restrictions on a patient despite the patient getting approval previously from a different doctor?

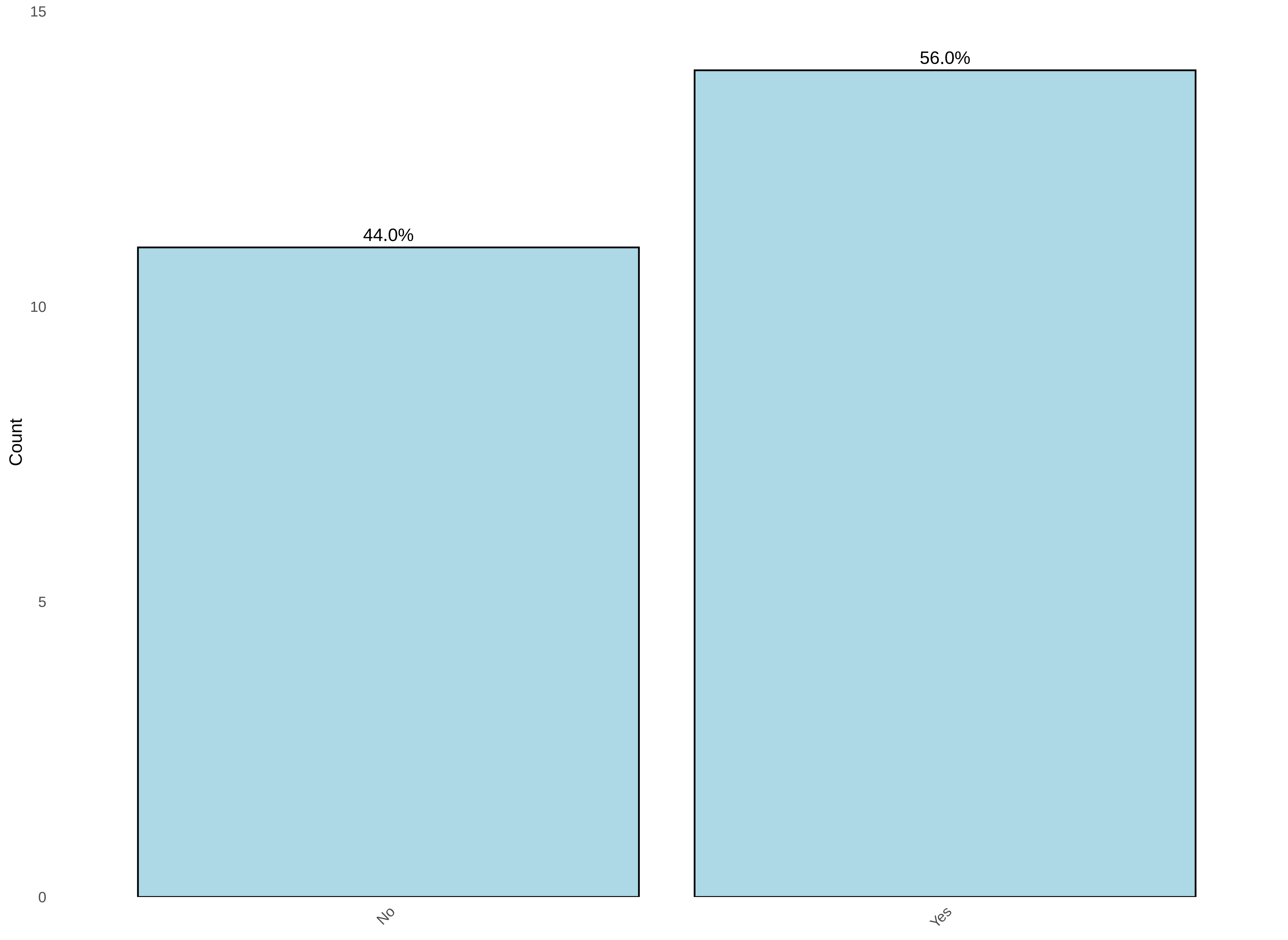

For determining fitness to drive: A comprehensive neurological history is necessary.

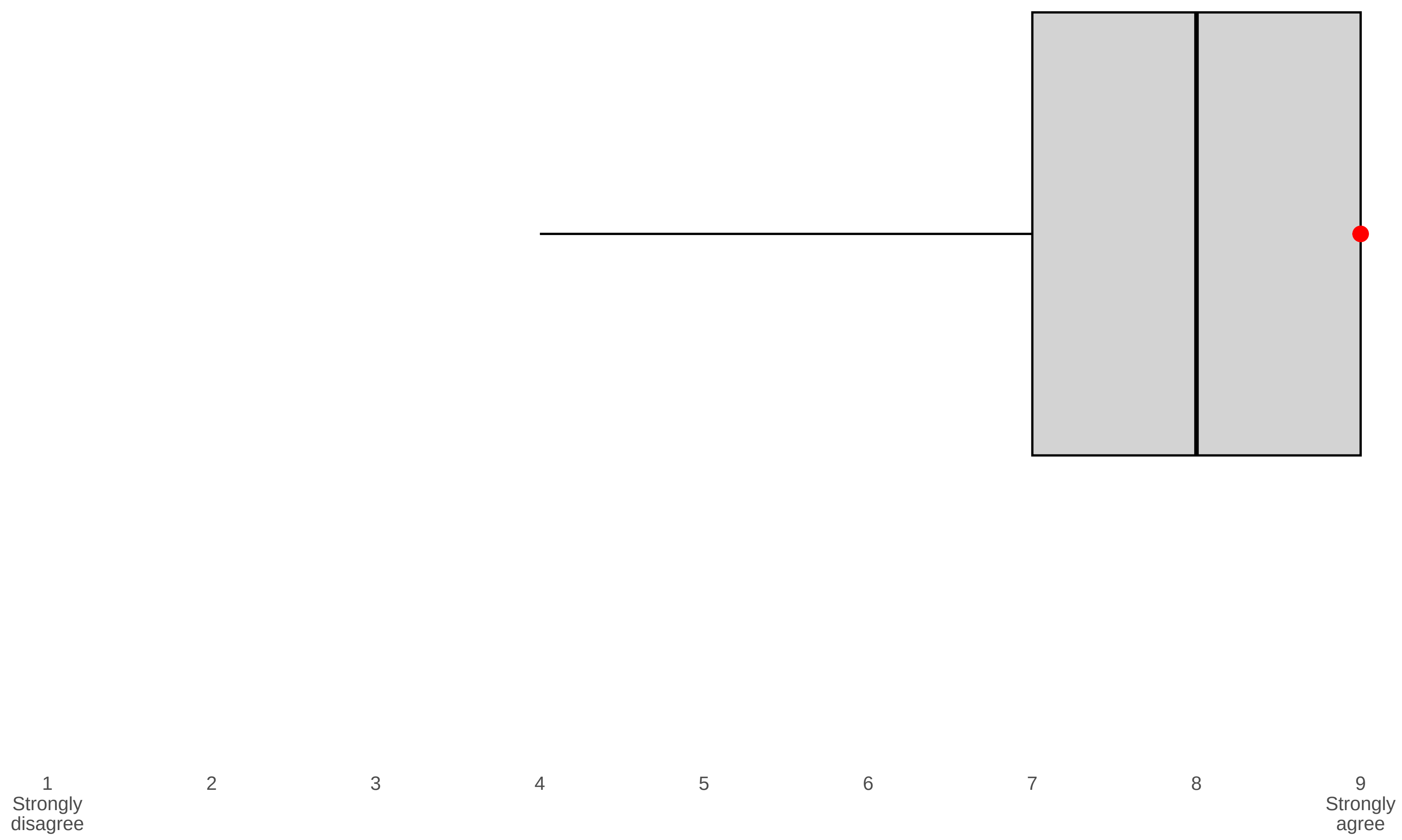

For determining fitness to drive: A neurological examination is necessary.

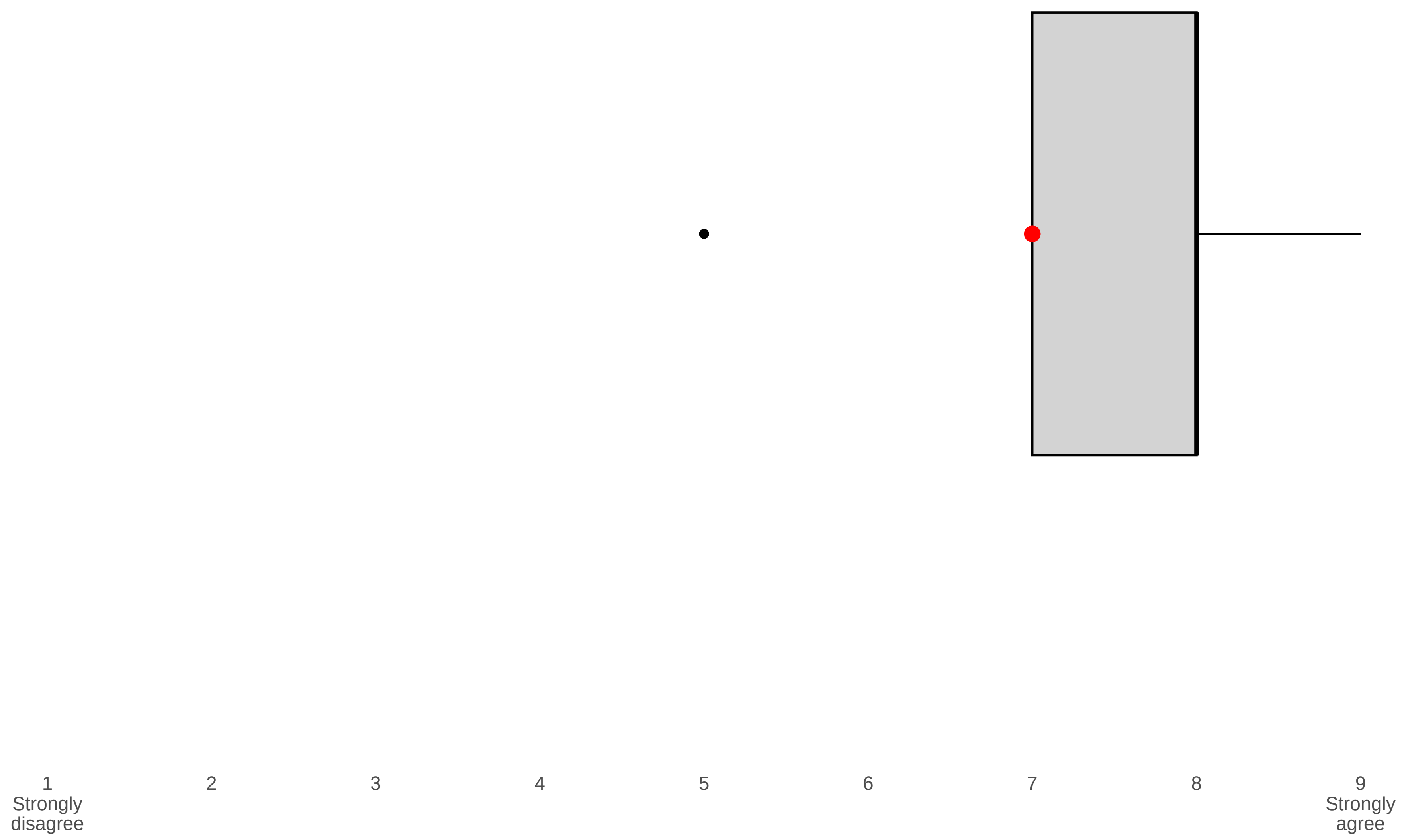

For determining fitness to drive: Baseline (at the time of initial driving assessment) visual fields are necessary.

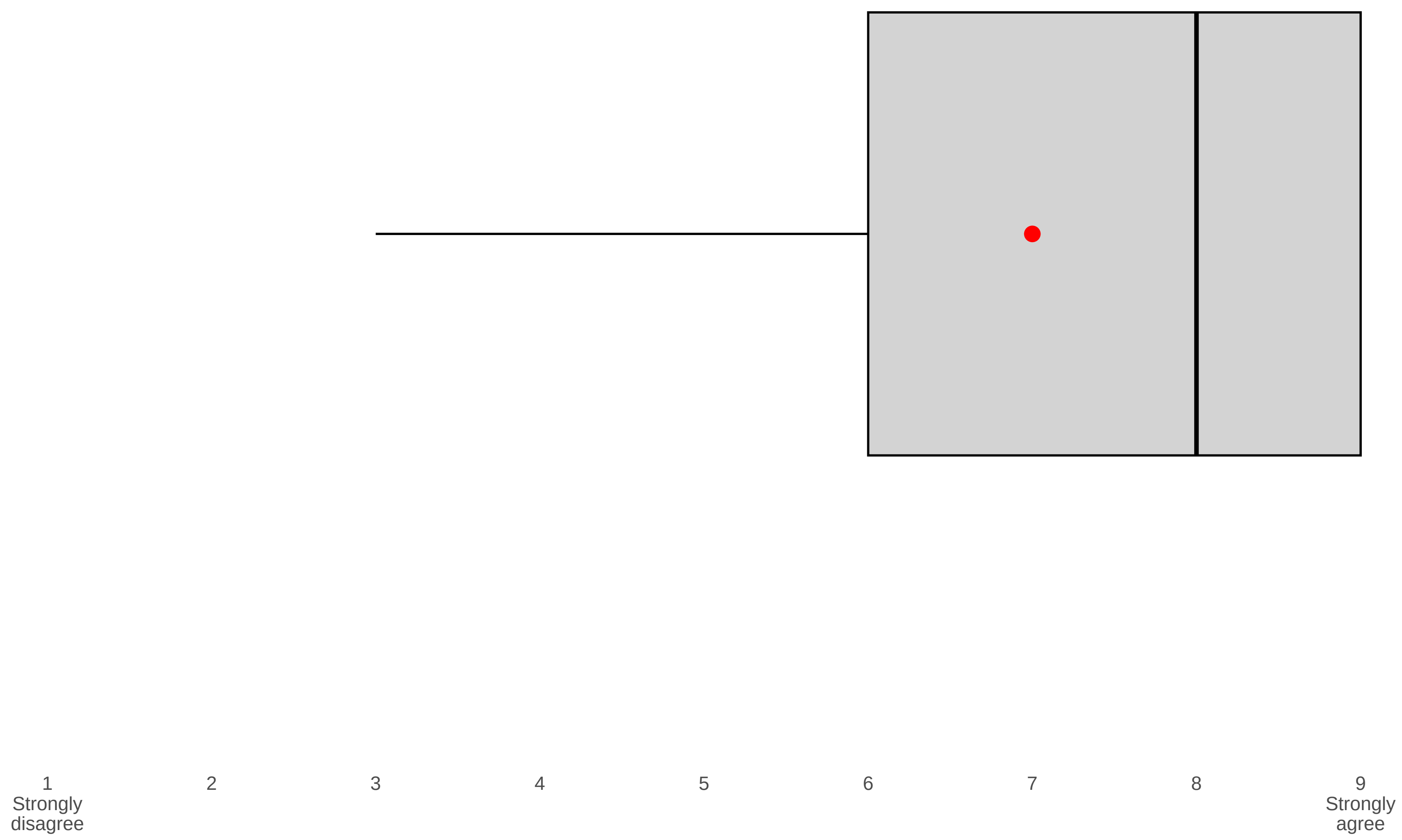

For determining fitness to drive: A formal visual assessment is necessary.

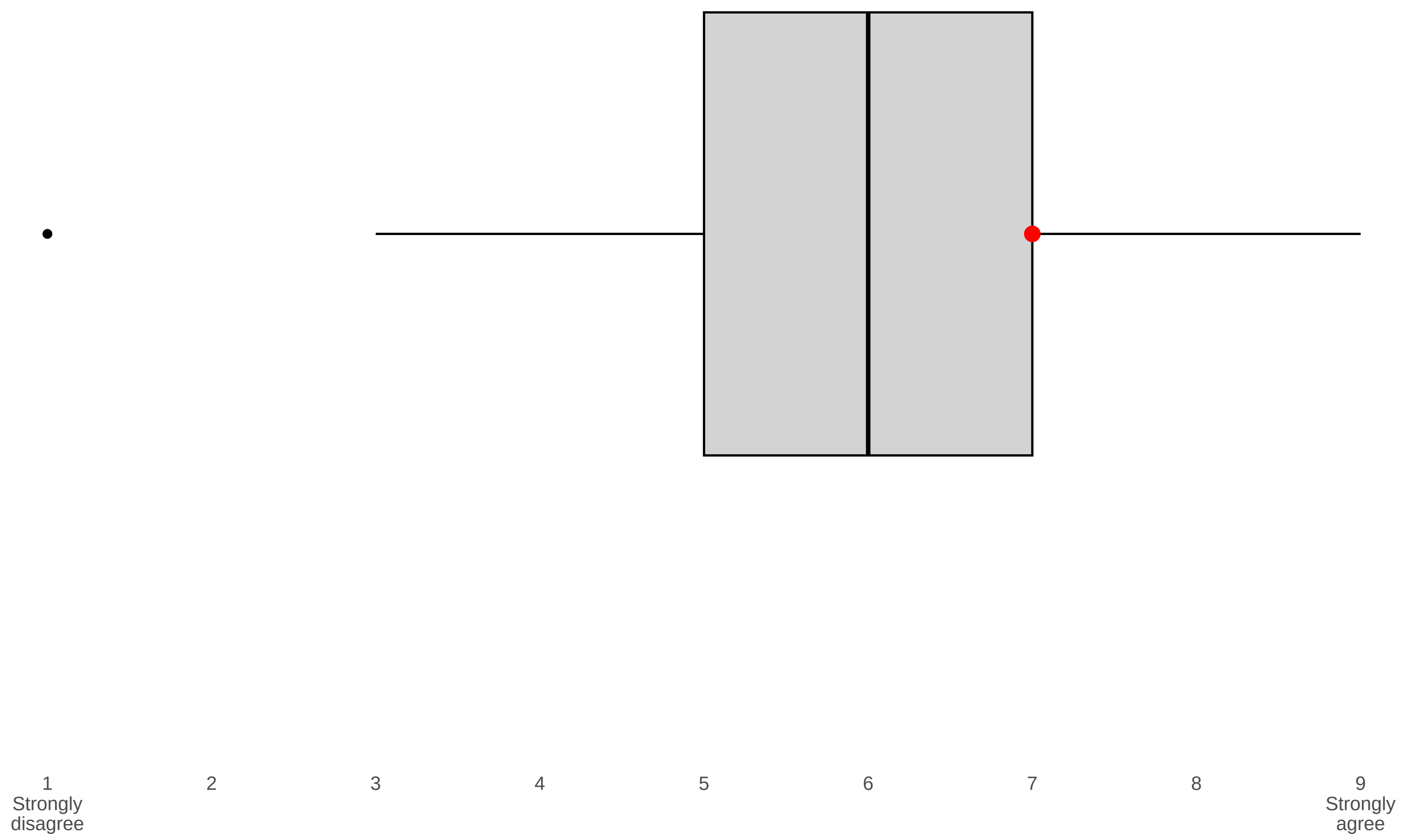

For determining fitness to drive: A baseline (at the time of initial driving assessment) neurological assessment with a focus on safe driving is necessary.

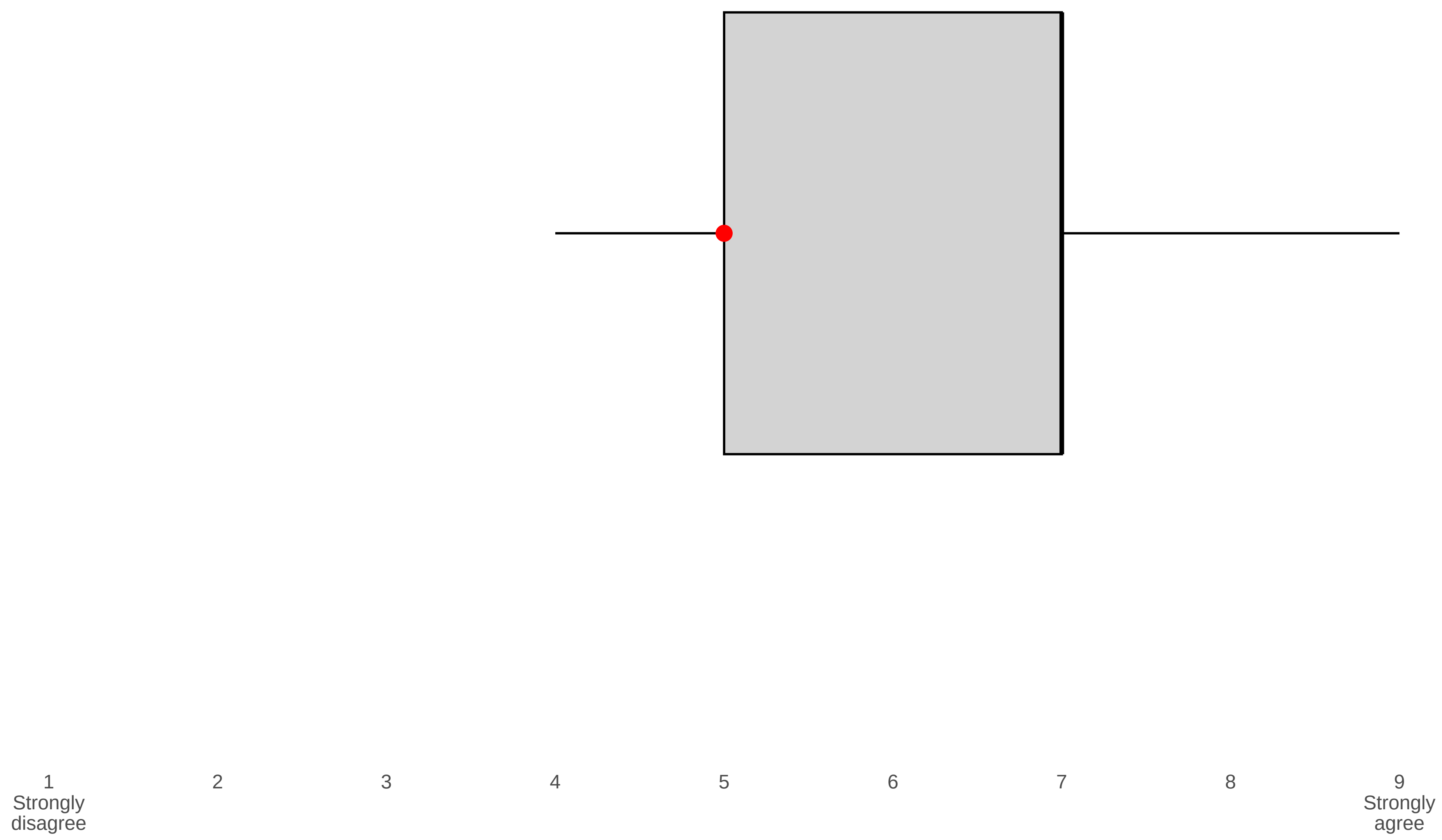

For determining fitness to drive: A defined seizure free period is necessary.

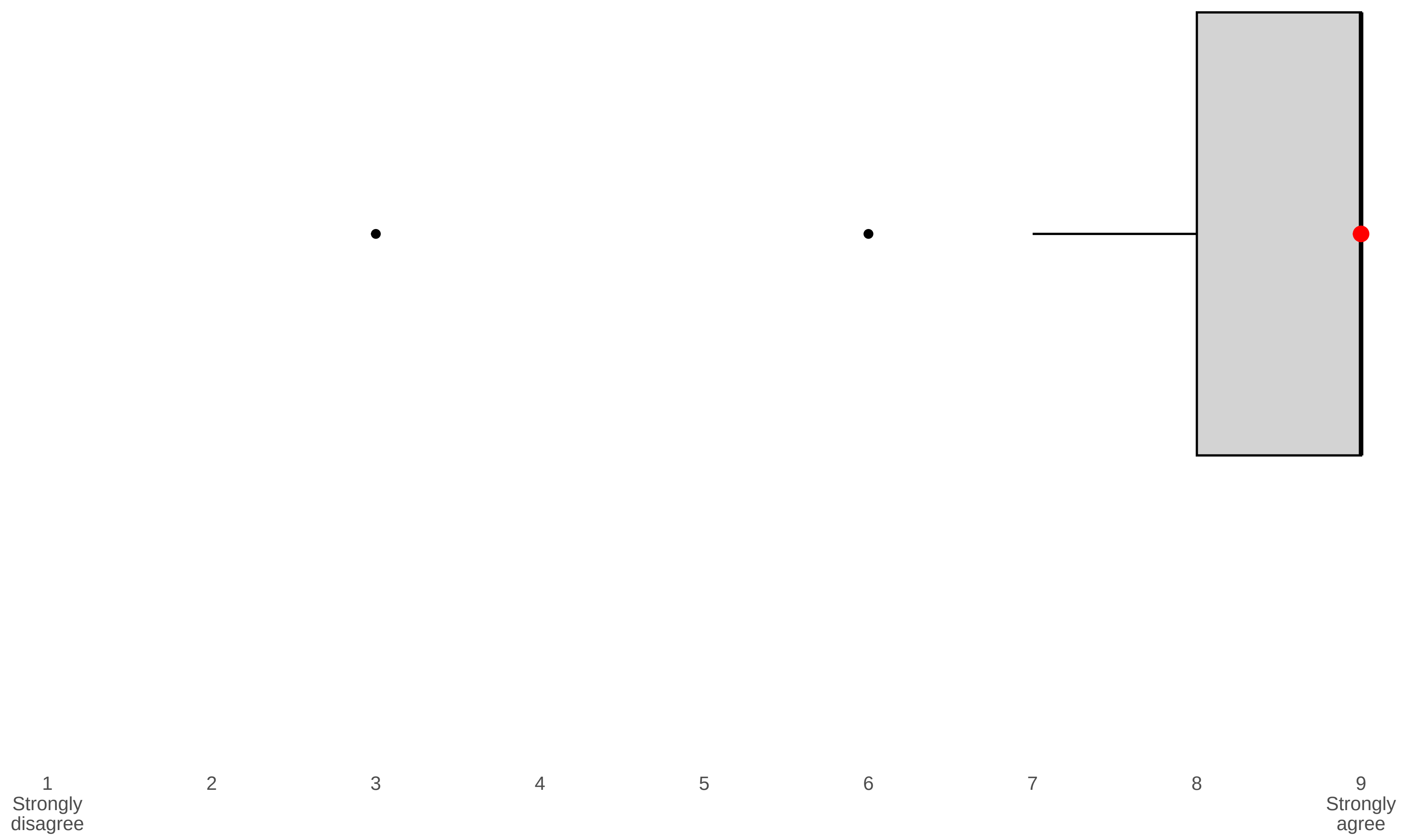

For determining fitness to drive: A stable repeat CT brain or MRI brain is necessary.

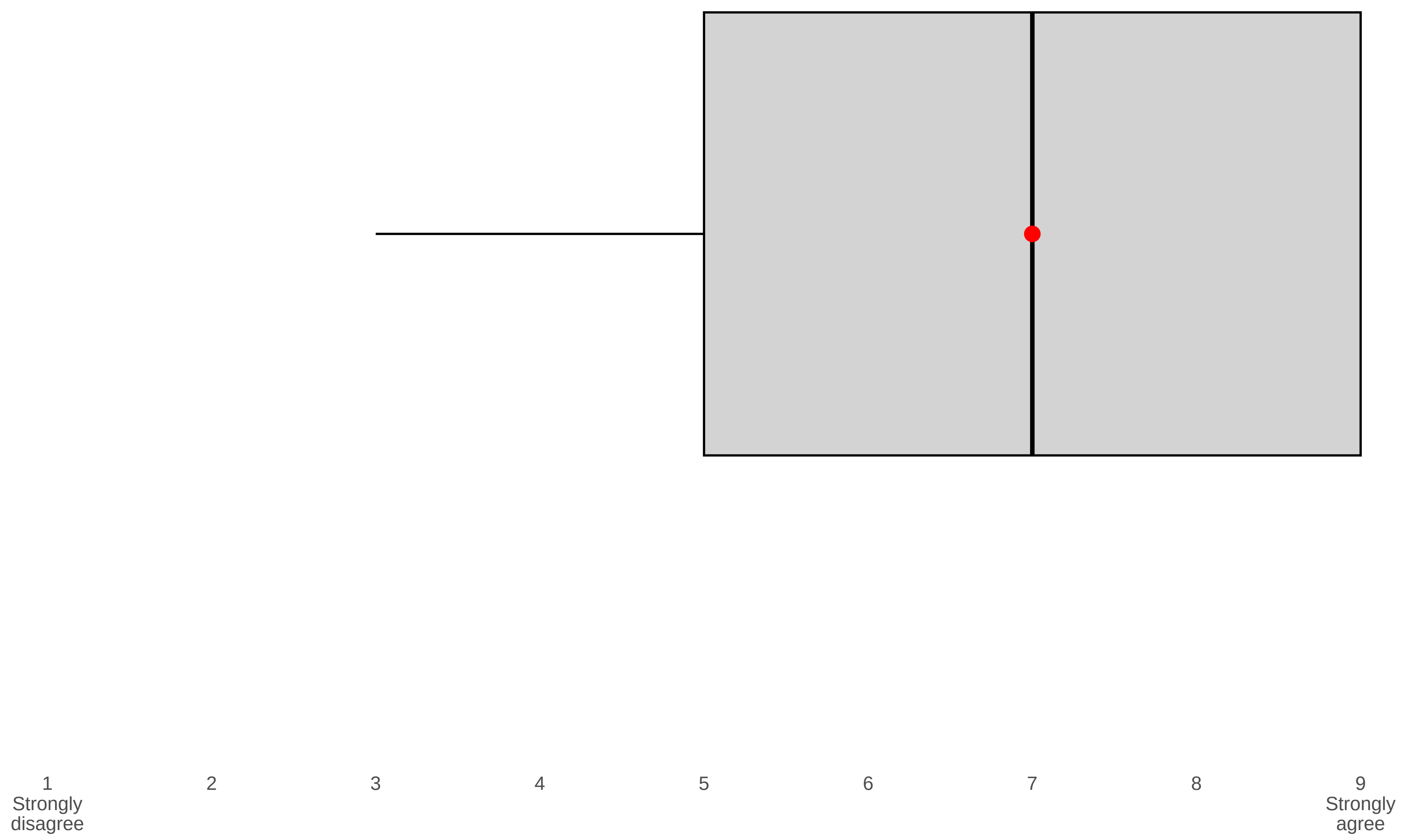

For determining fitness to drive: A driving assessment with a driving instructor / occupational therapist is necessary.

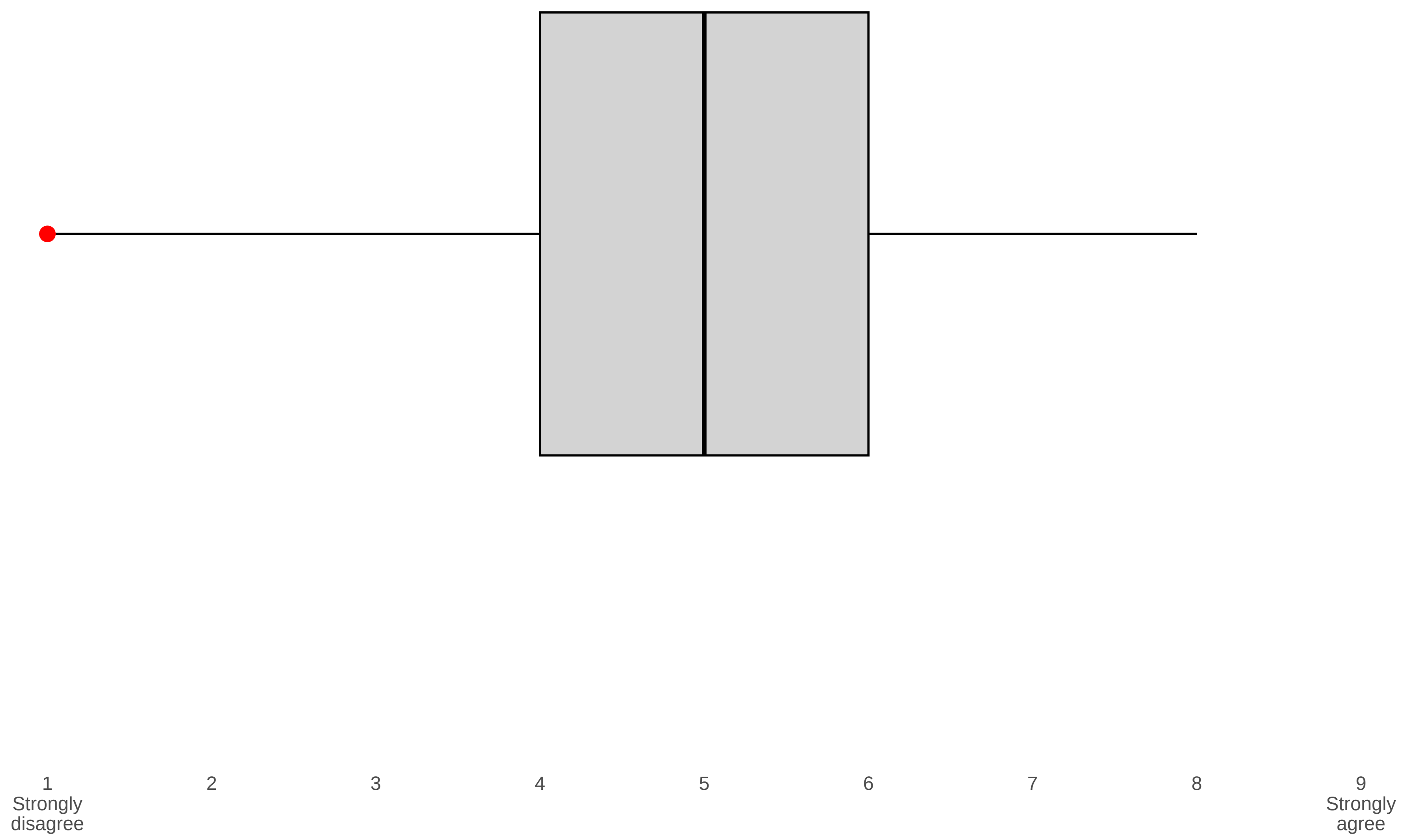

A defined period post−operatively if relevant is necessary.

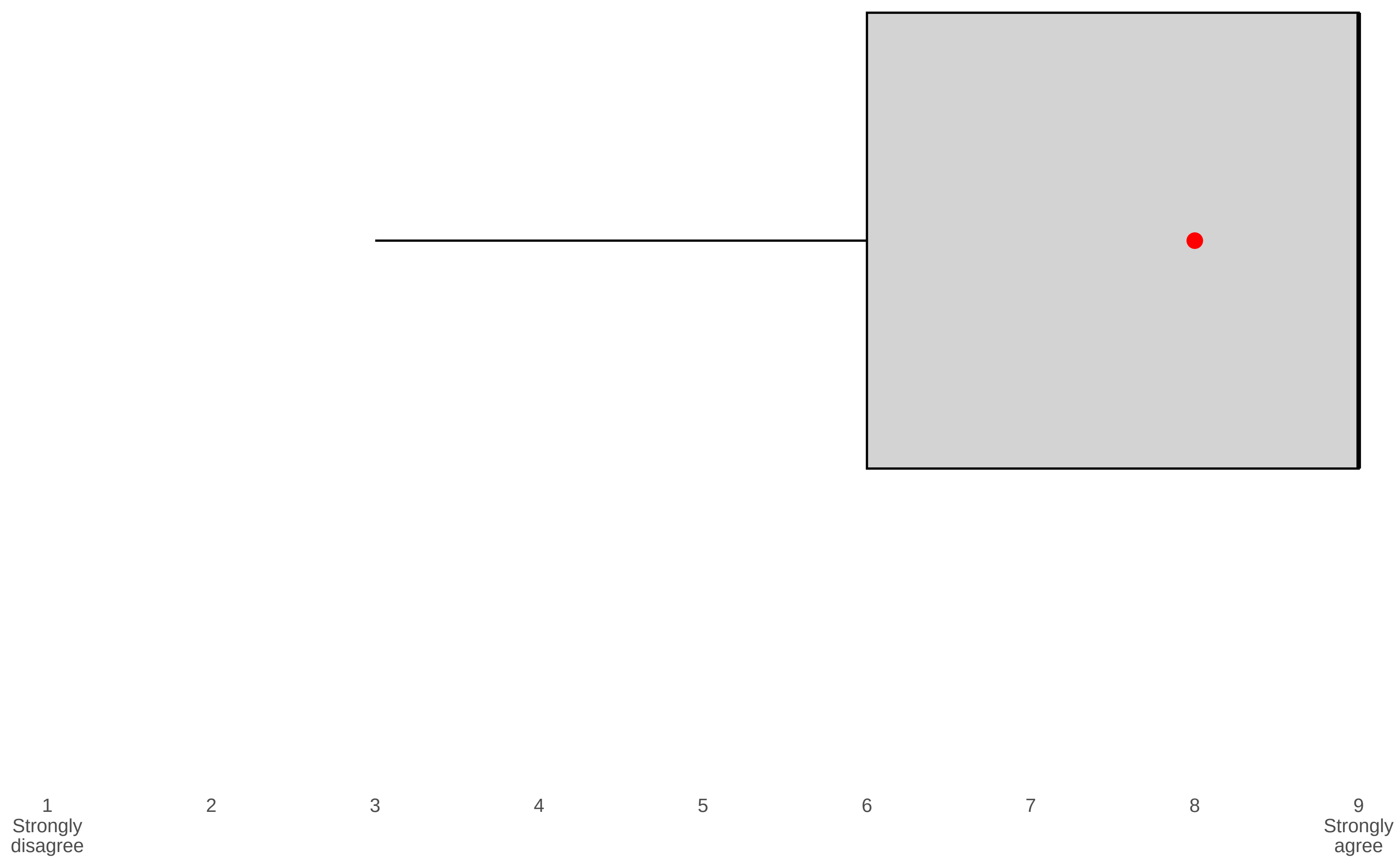

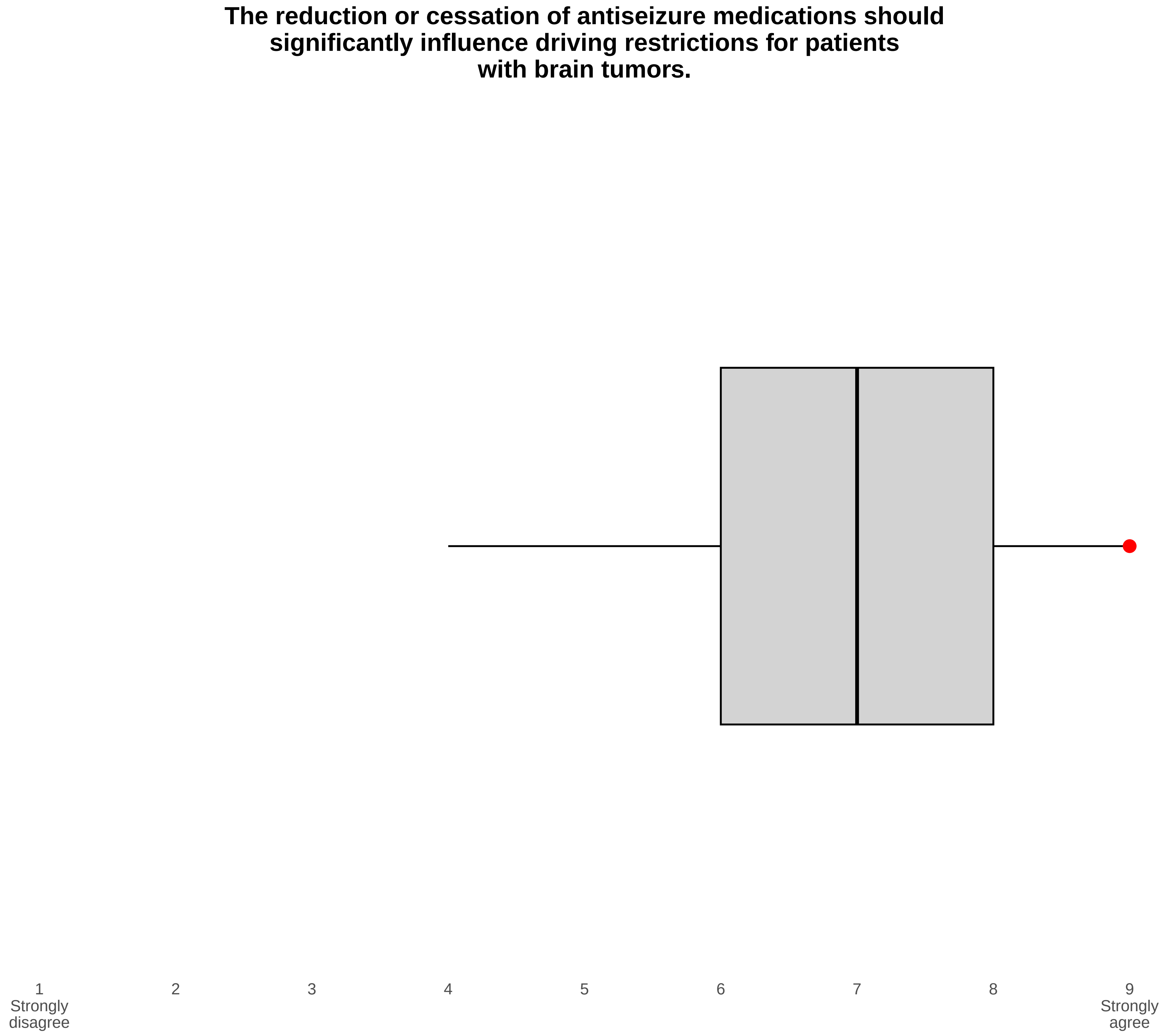
The reduction or cessation of antiseizure medications should significantly influence driving restrictions for patients with brain tumors.
1 Strongly disagree
2
3
4
5
6
7
8
9 Strongly agree

The status of active treatment or the time since the last treatment should significantly impact driving restrictions for patients with brain tumors.

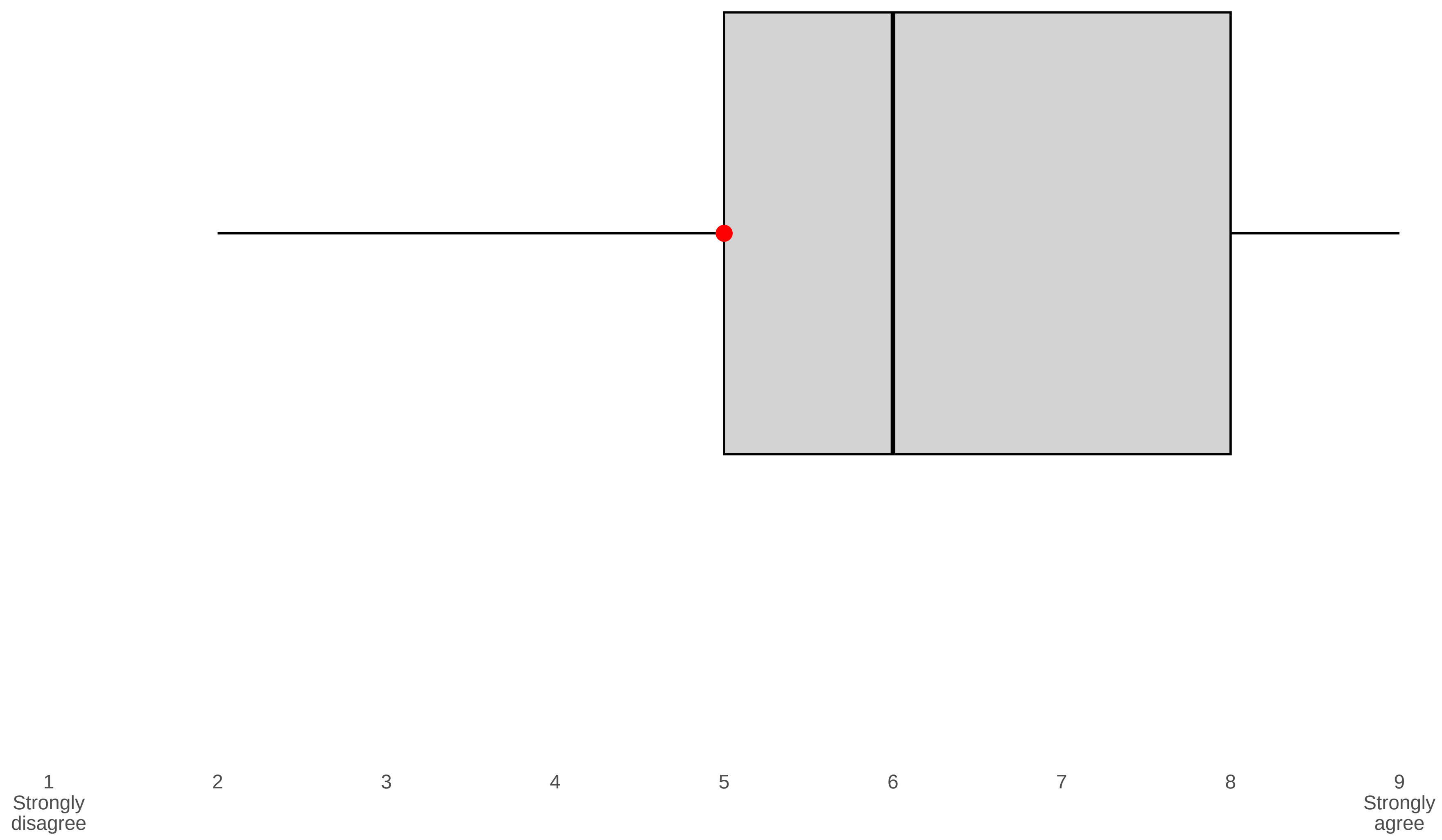

I think it is important to continually reassess fitness to drive.

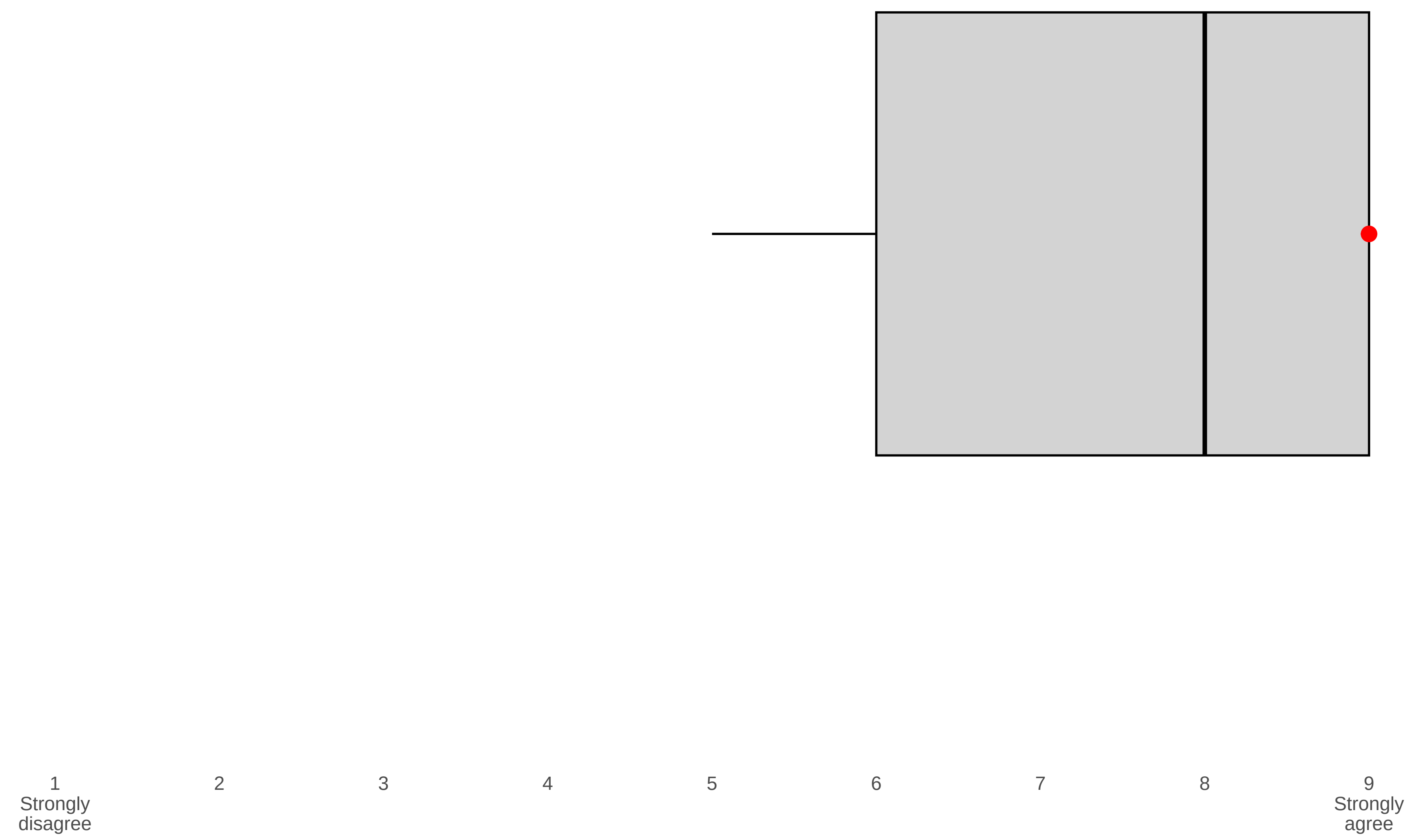

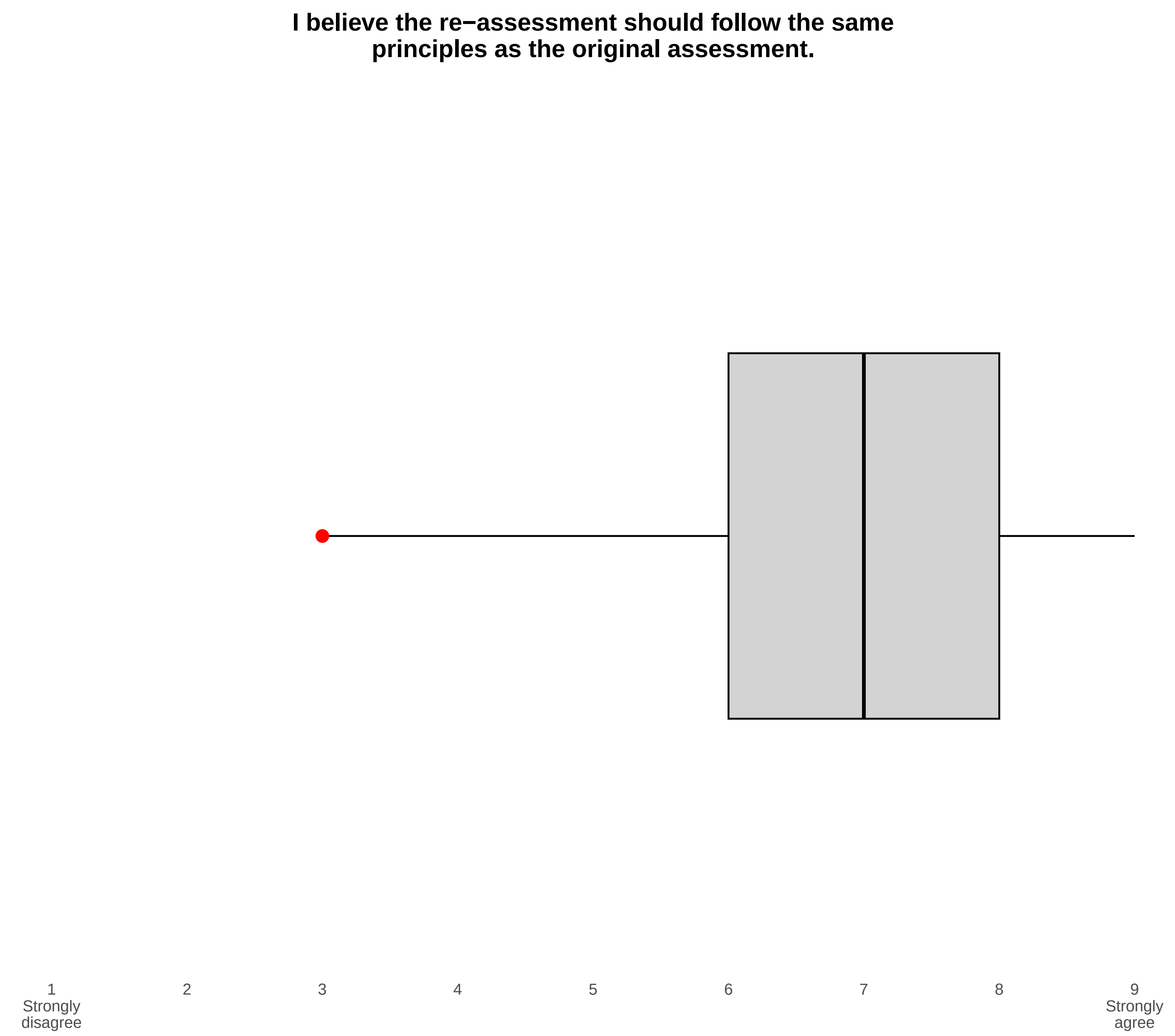
I believe the re−assessment should follow the same principles as the original assessment.
1
Strongly disagree
2
3
4
5
6
7
8
9
Strongly agree

Patients should sign a document with the recommendation of their physician.

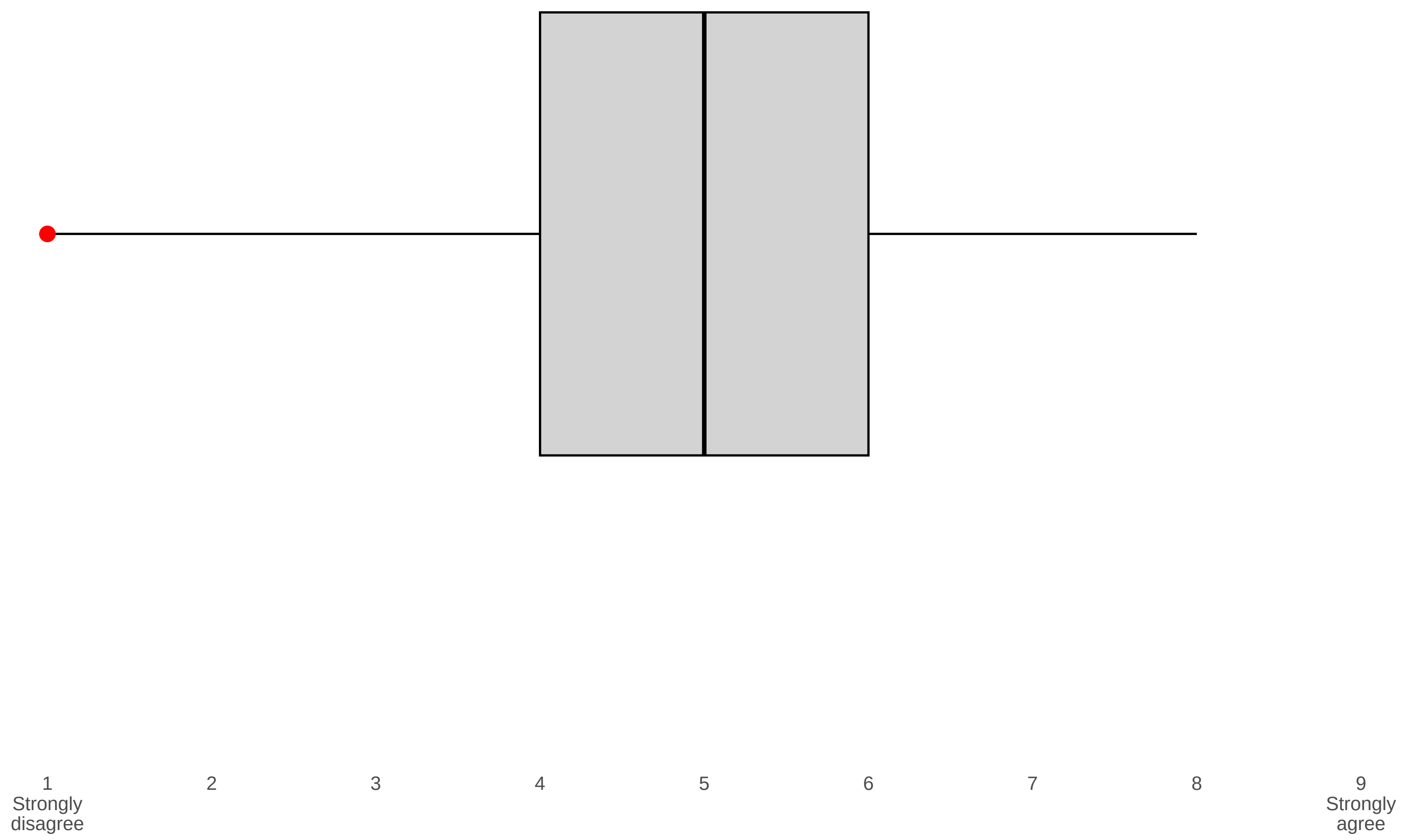

The current epilepsy guidelines for seizure−free period are adequate for patients with brain tumours / following intracranial surgery.

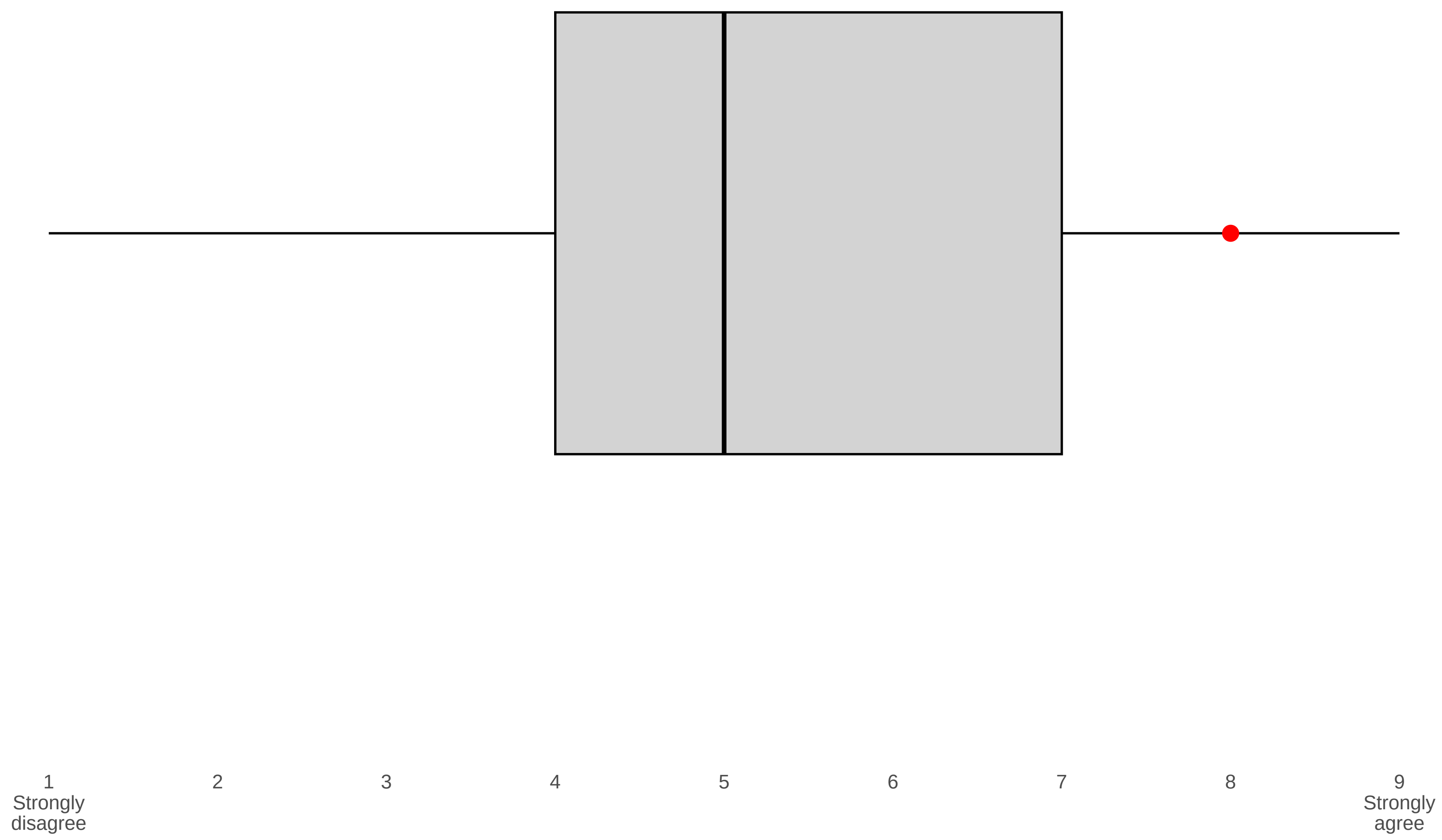

Each brain tumour subtype needs its own specific recommendation.

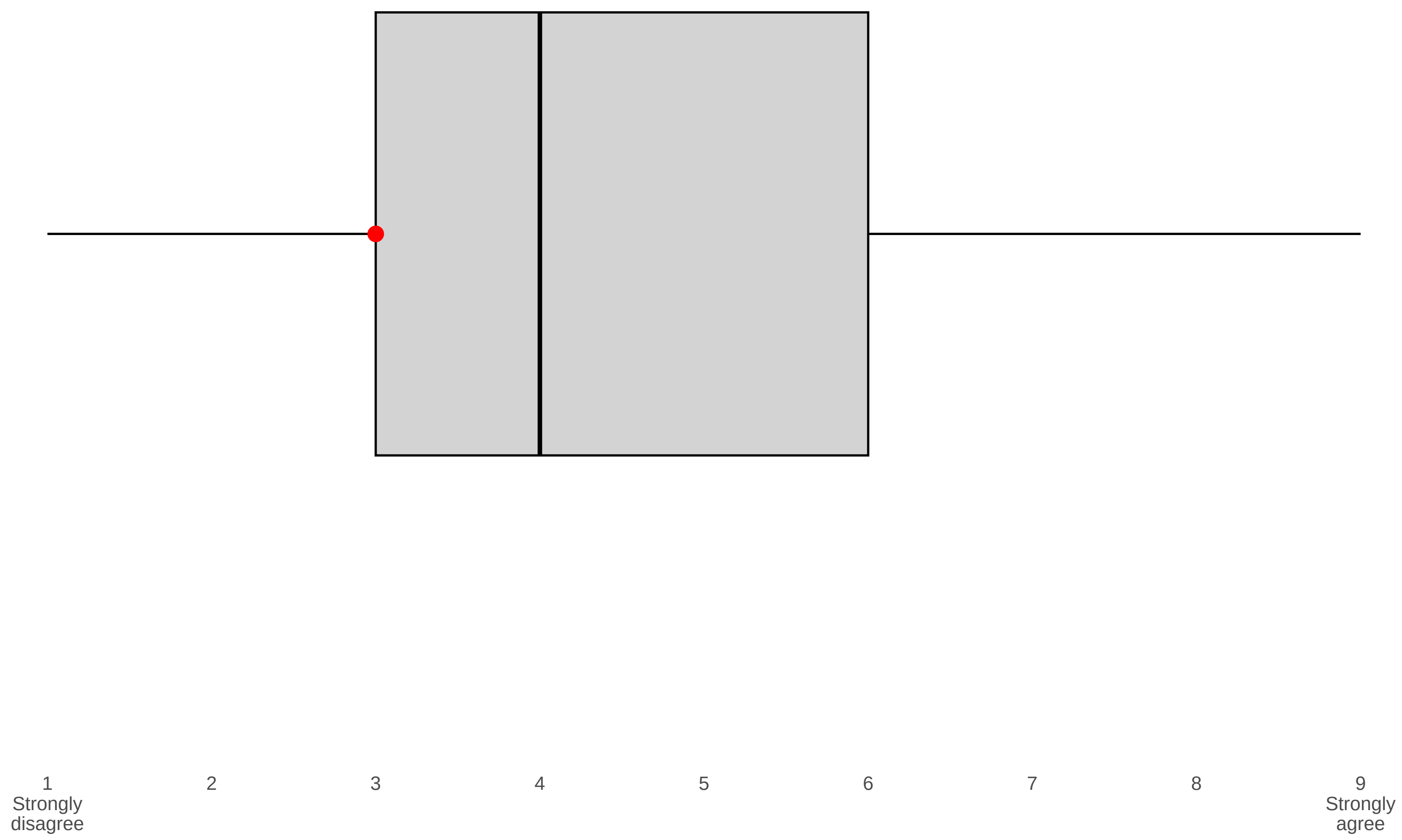

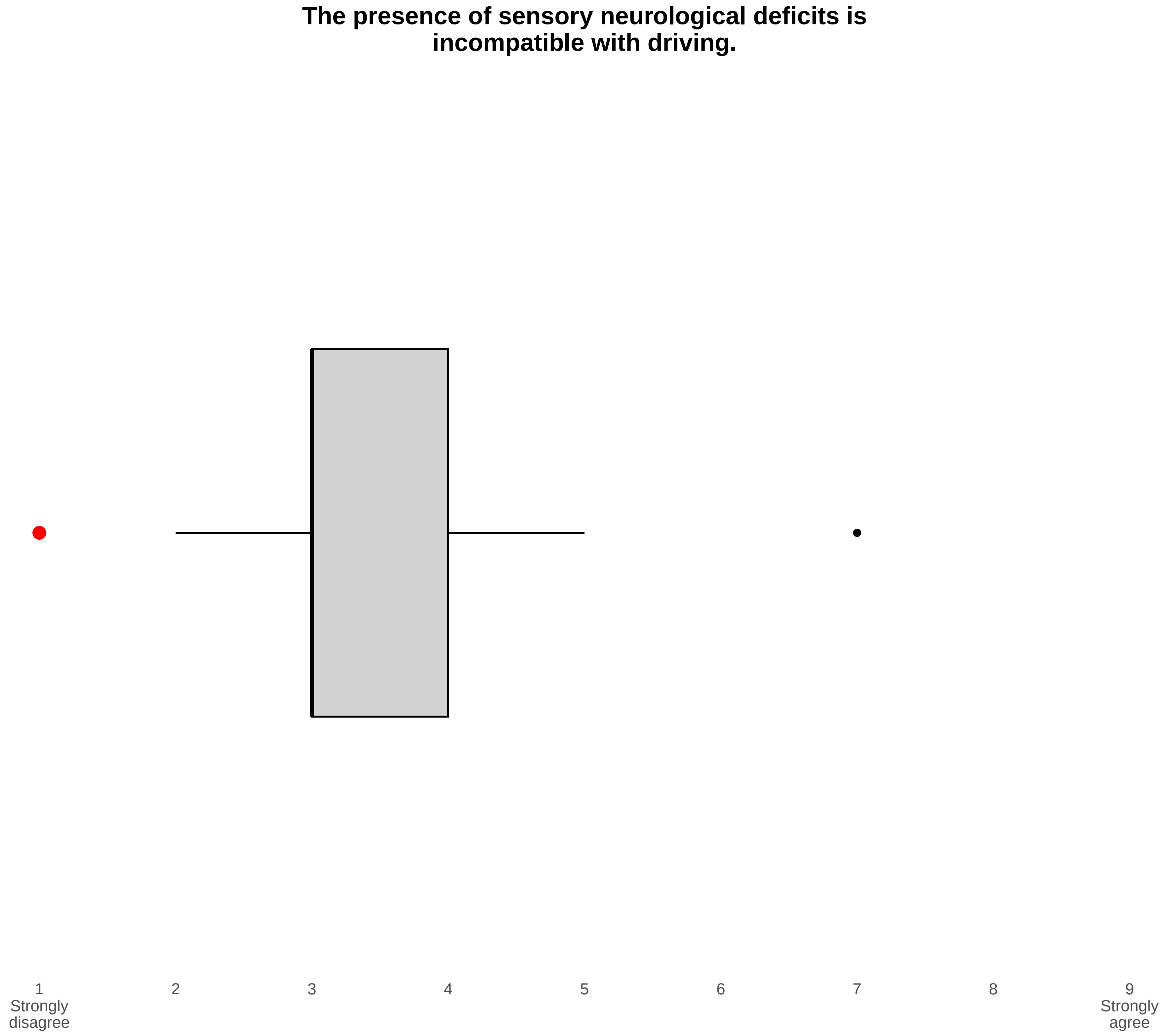
The presence of sensory neurological deficits is incompatible with driving.
1
Strongly disagree
2
3
4
5
6
7
8
9
Strongly agree

The presence of motor neurological deficits is incompatible with driving.

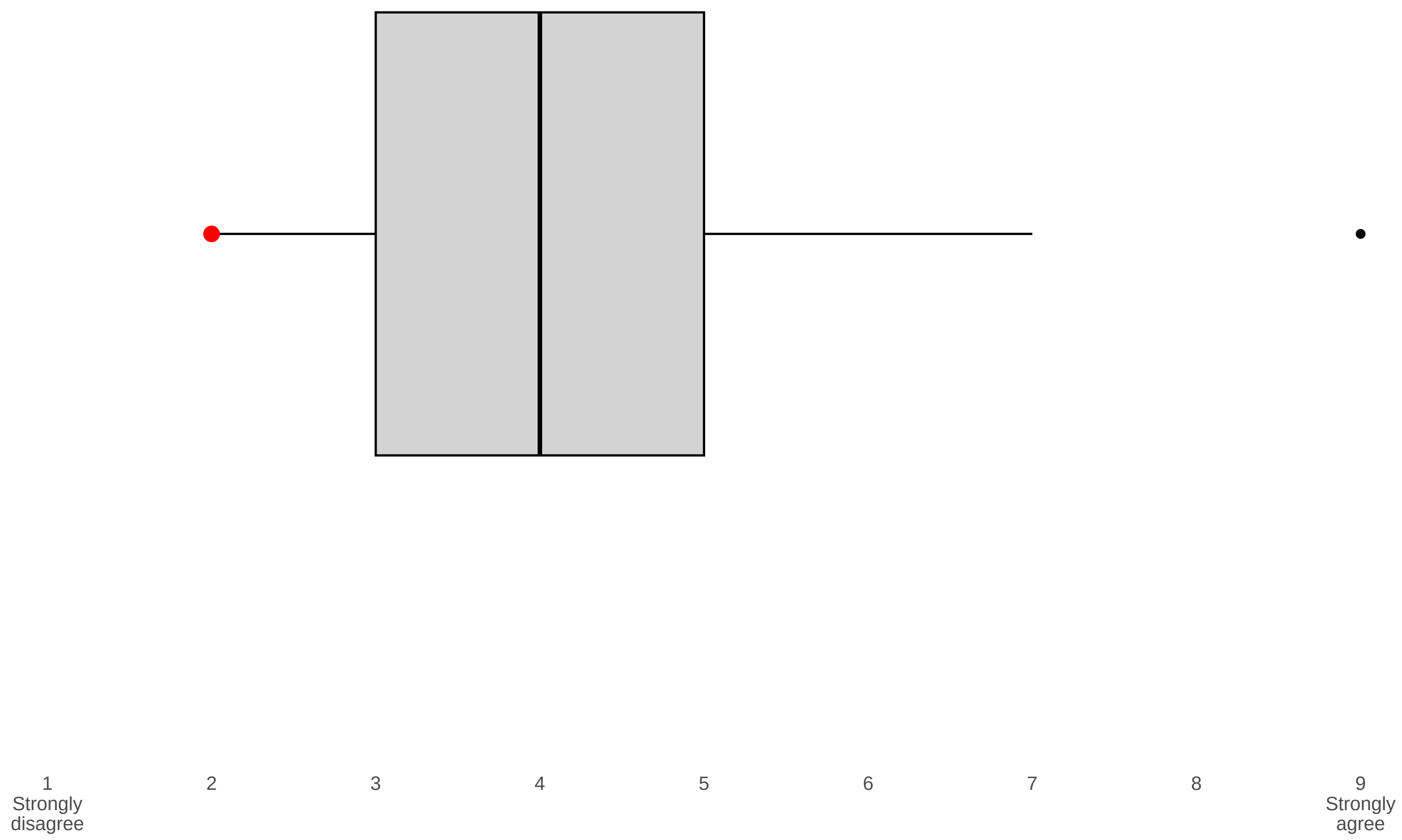

The presence of cognitive neurological deficits is incompatible with driving.

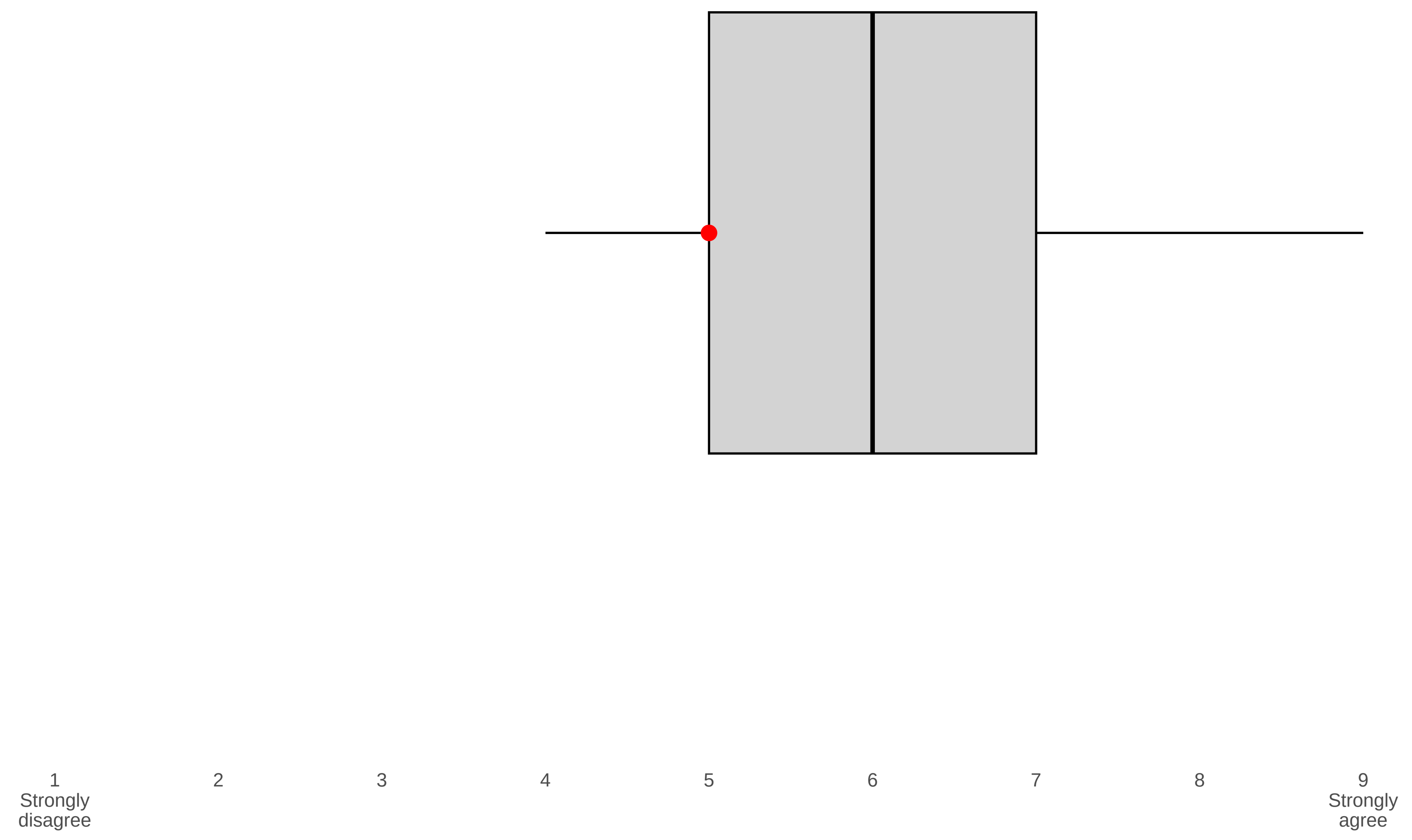

The presence of an EEG with epilepsy−specific potential is incompatible with driving.

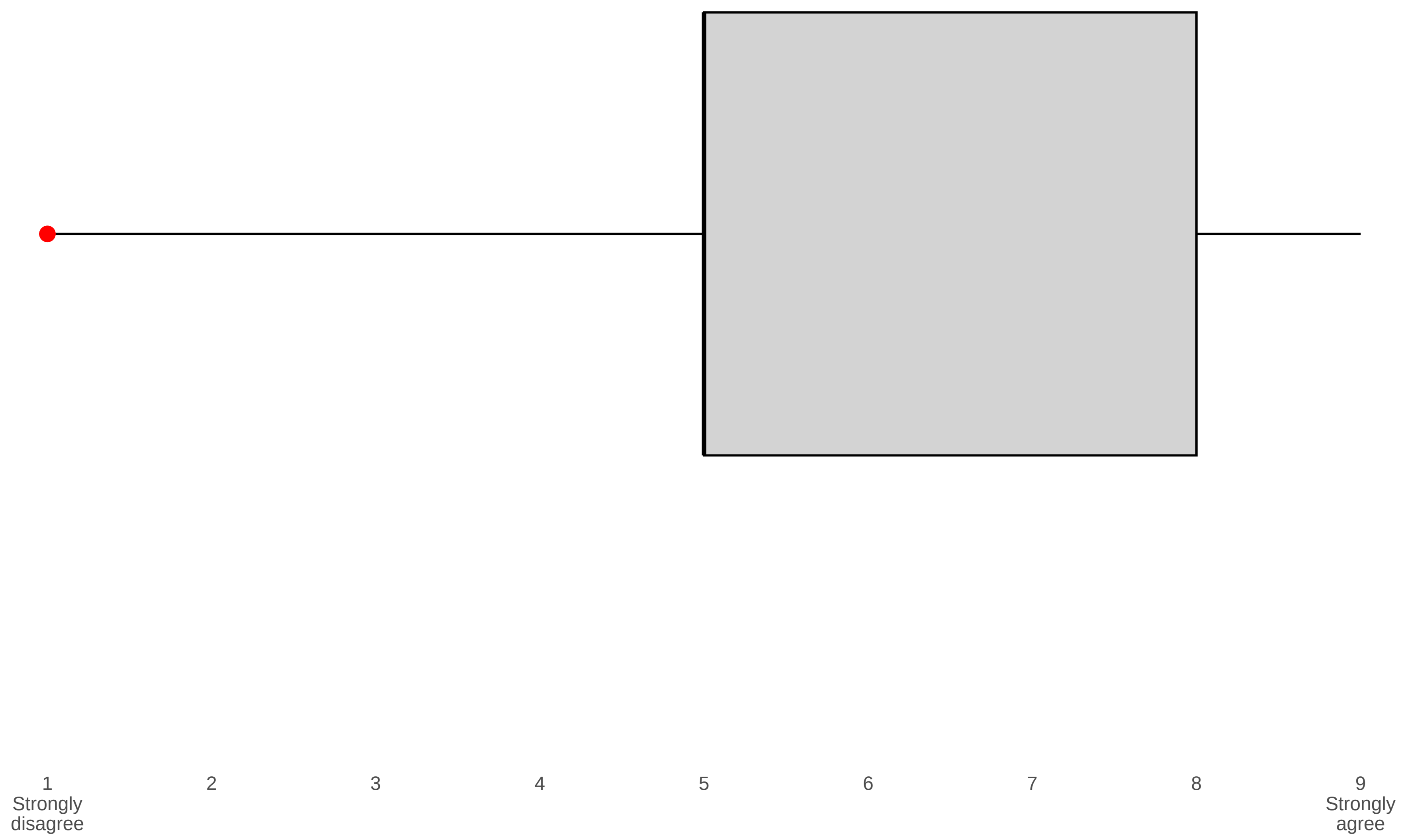

The use of anti−epileptic medications is incompatible with driving.

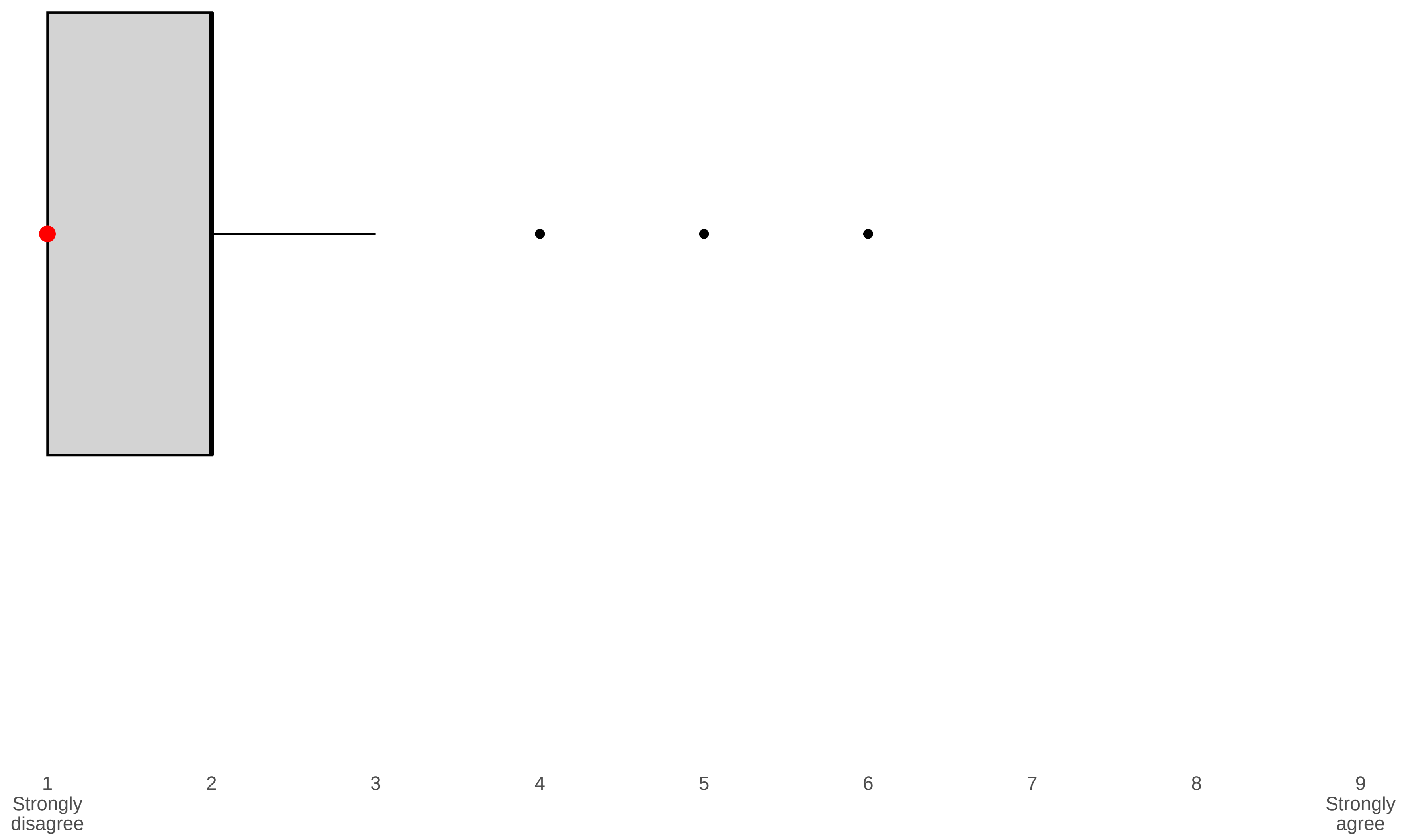

The presence of ongoing seizures is incompatible with driving.

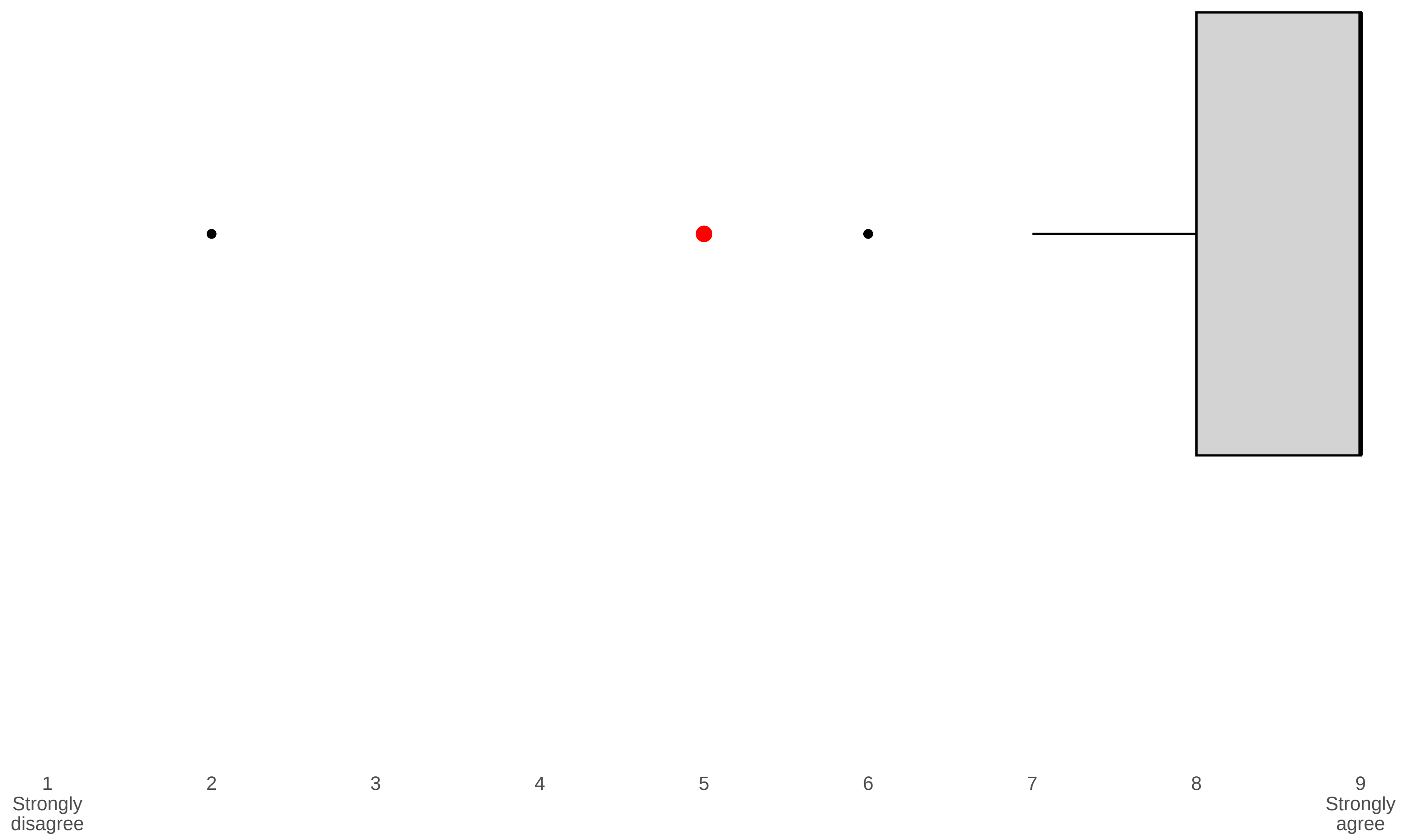

The presence of superior quadrantanopia is incompatible with driving.

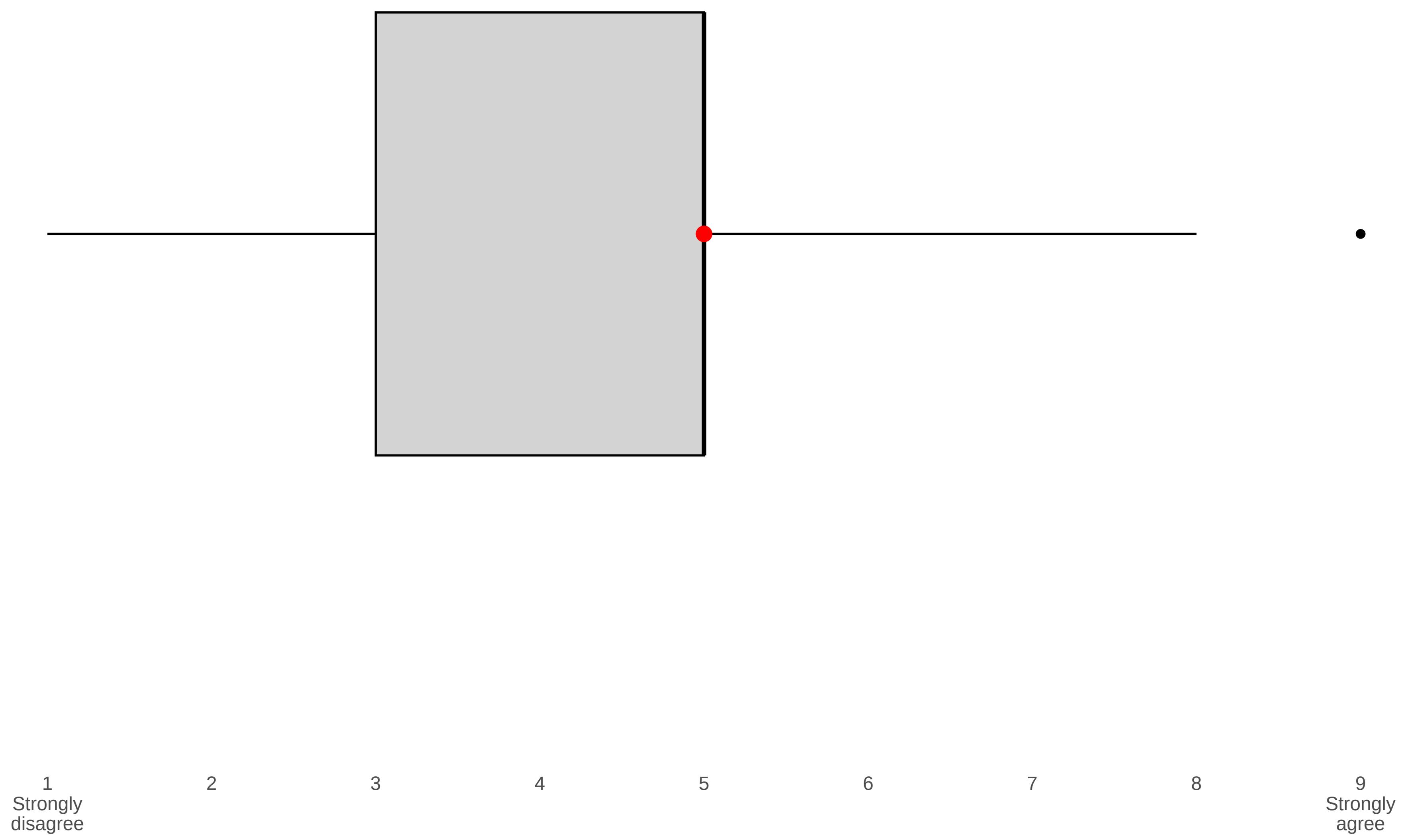

The presence of inferior quadrantanopia is incompatible with driving.

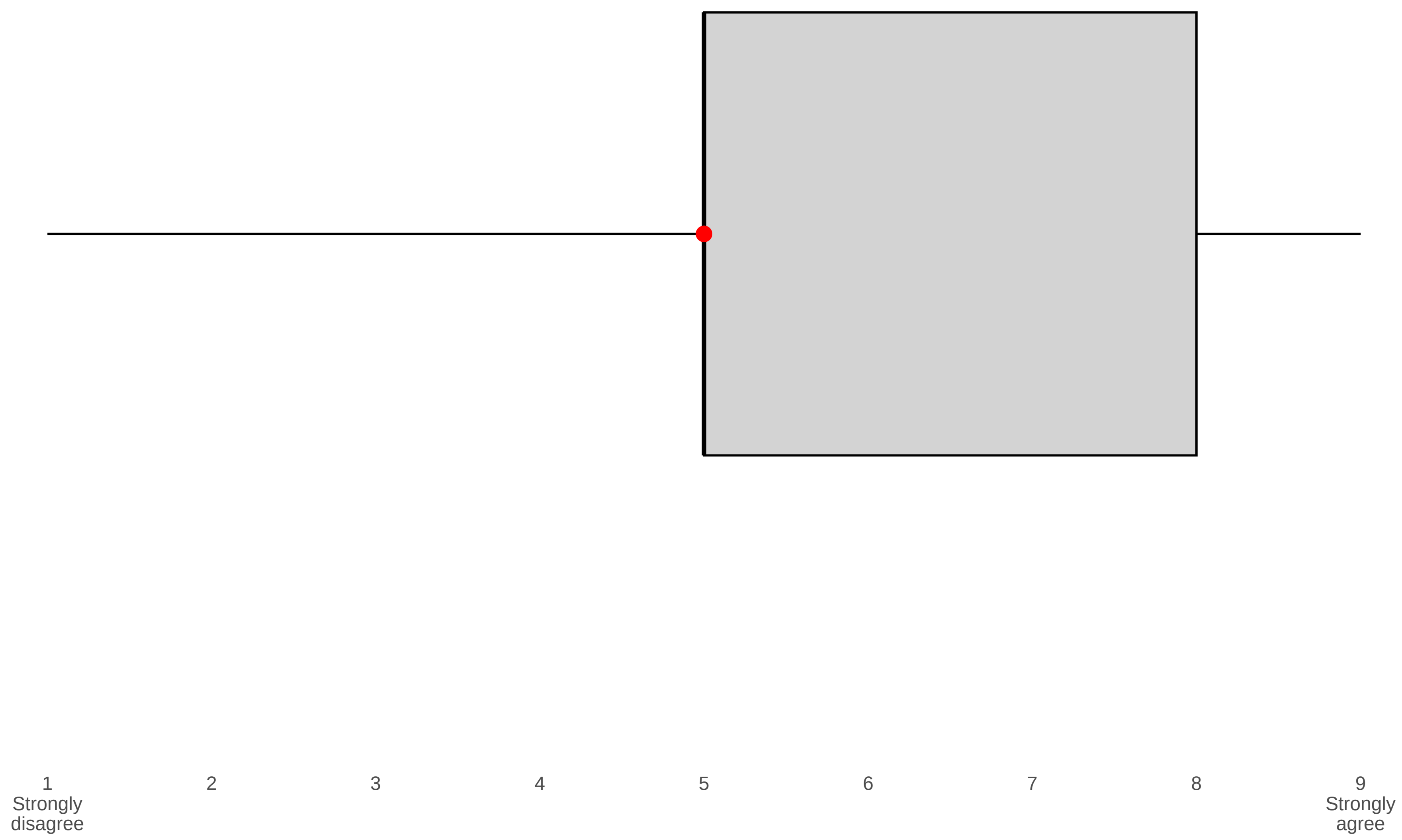

The presence of hemianopia is incompatible with driving.

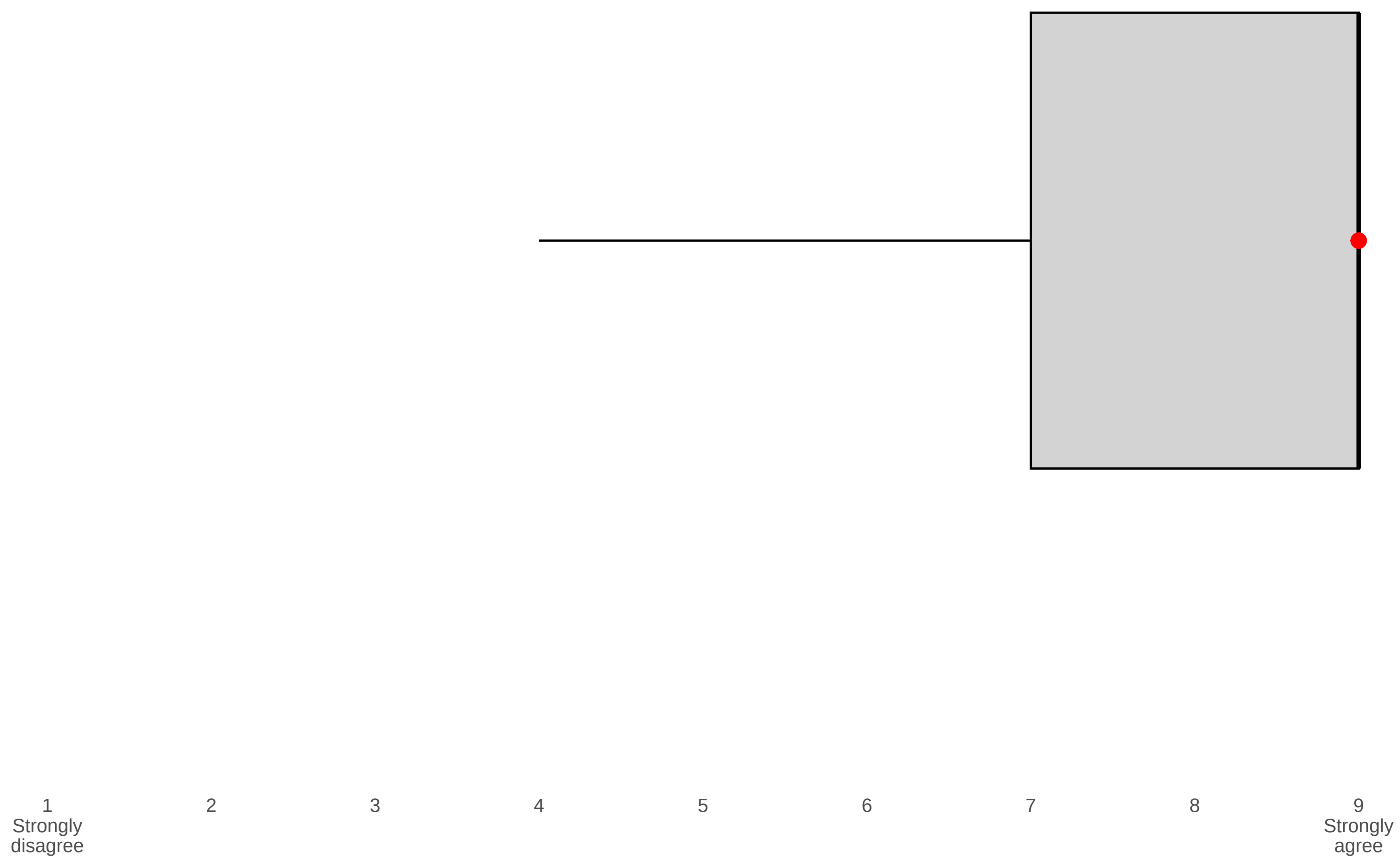

Impaired executive function is incompatible with driving.

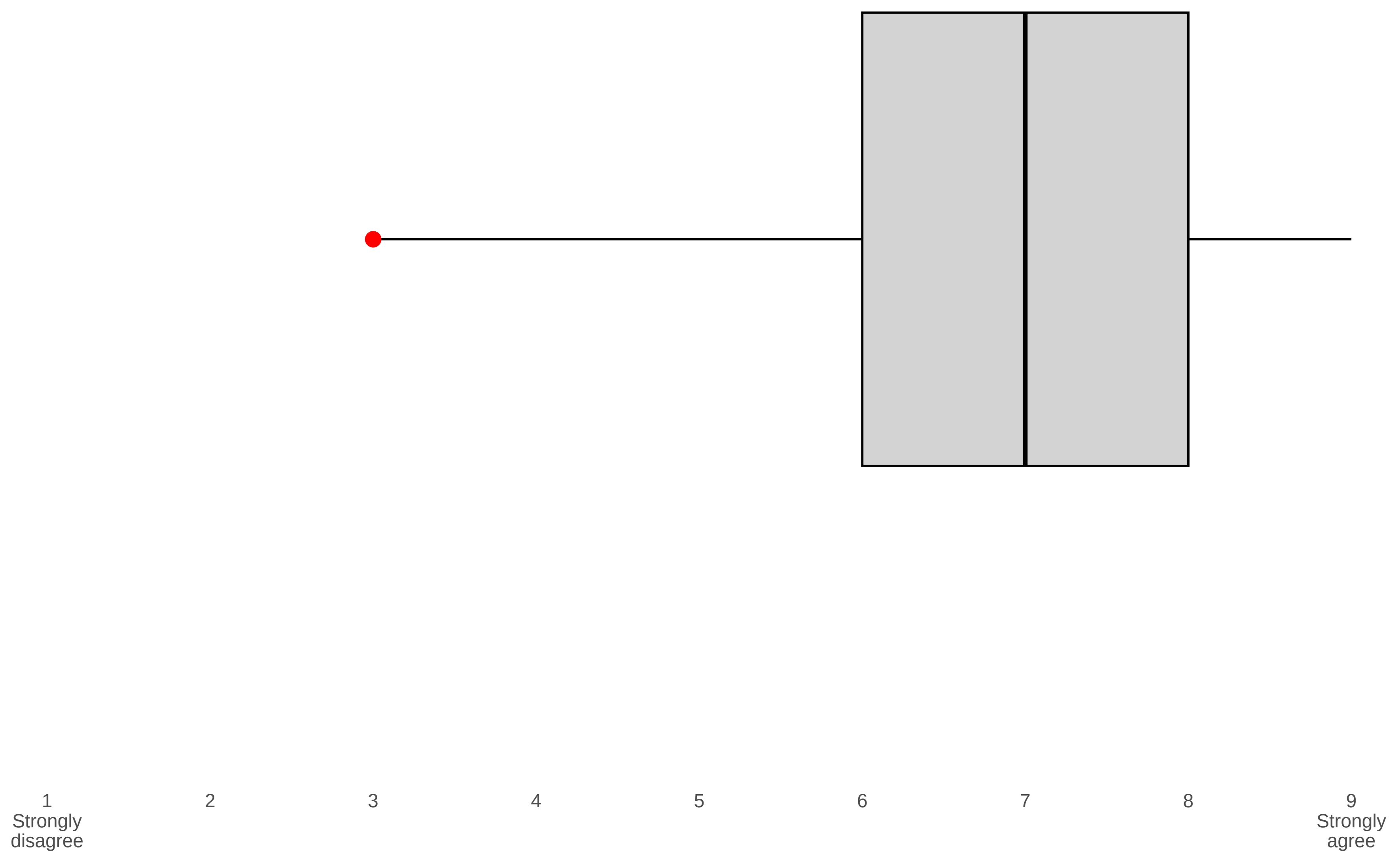

Impaired visuospatial function is incompatible with driving.

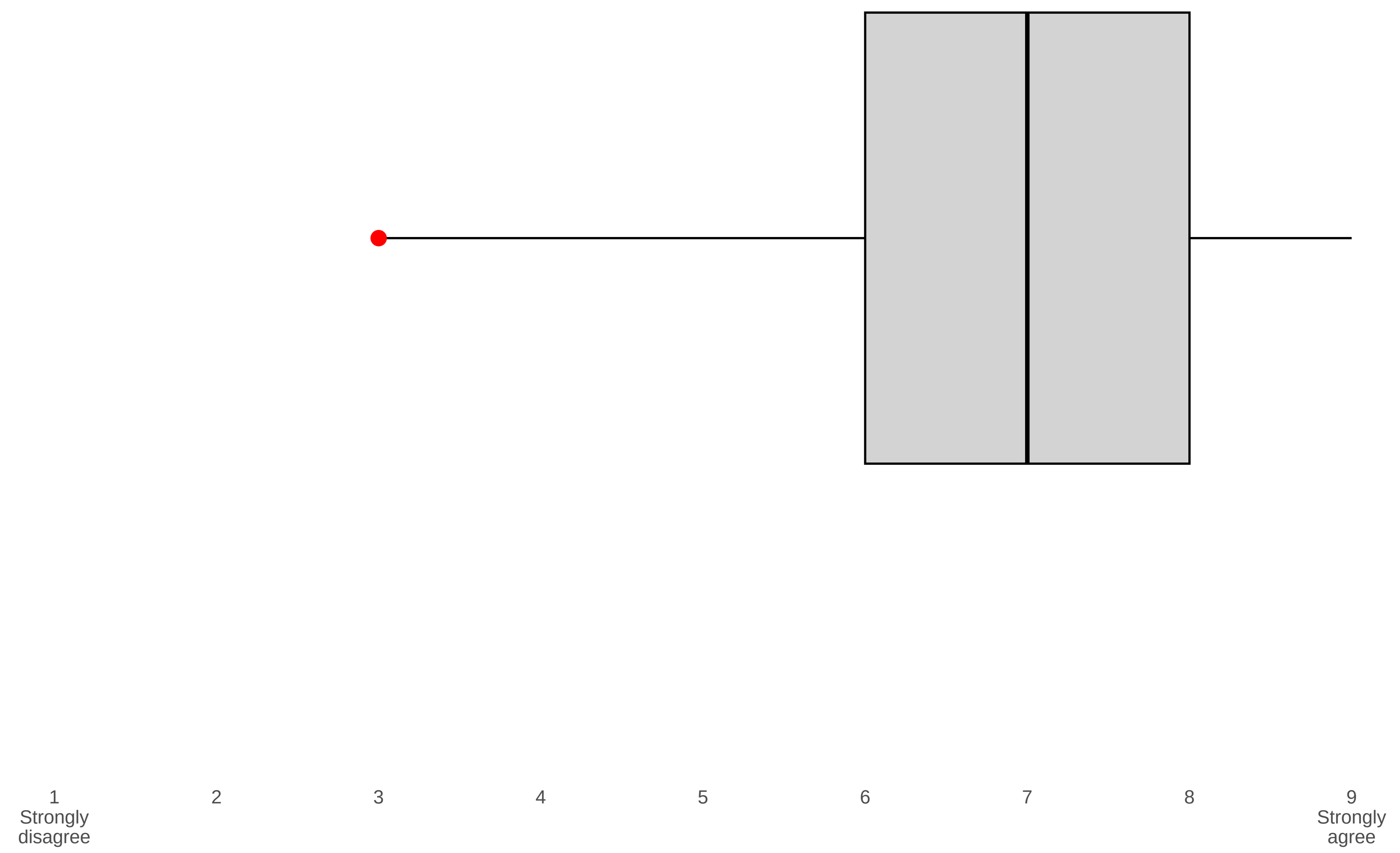

Reduced speed of information processing is incompatible with driving.

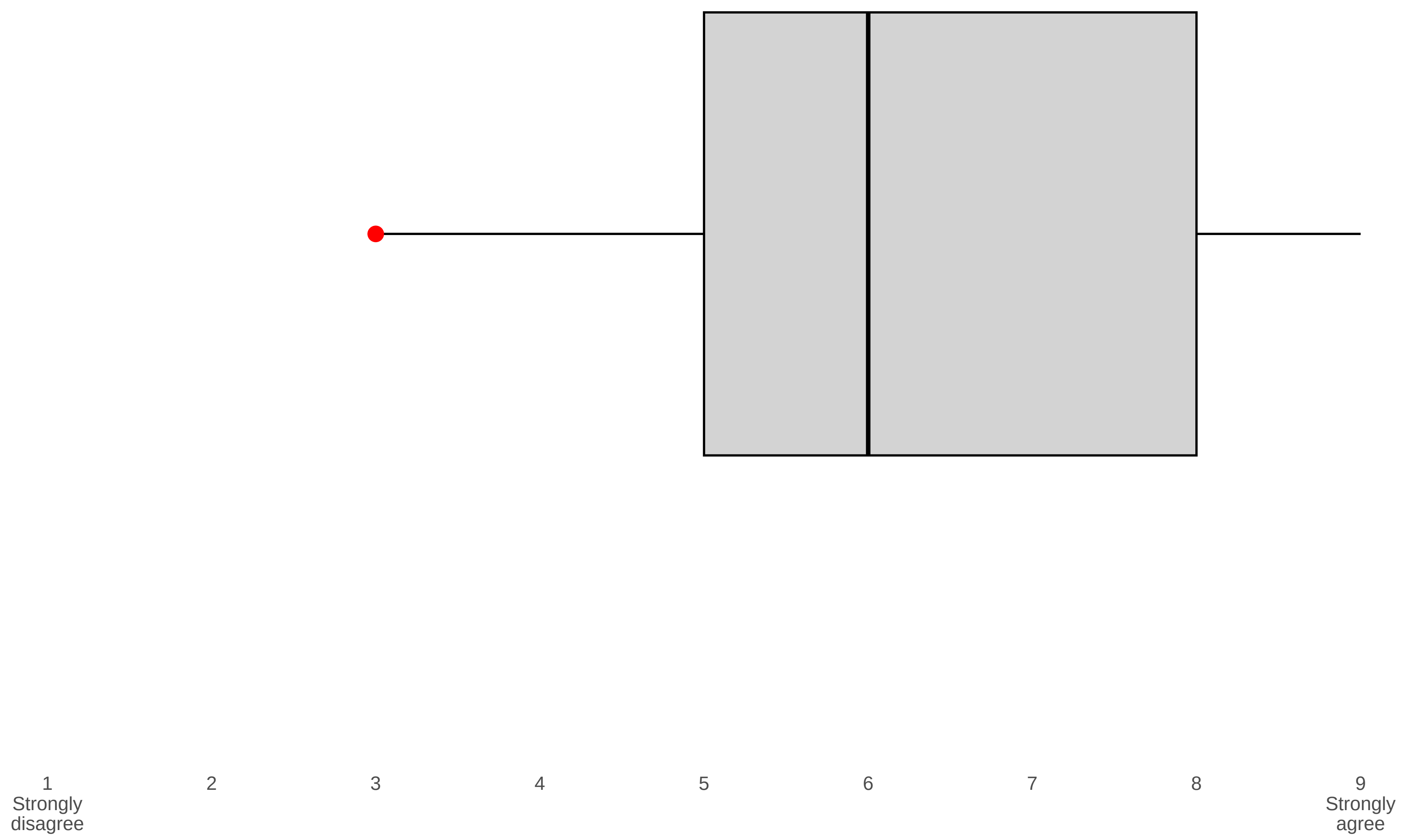

Reduced mini−mental state examination (MMSE) with a score of 25−29 is incompatible with driving.

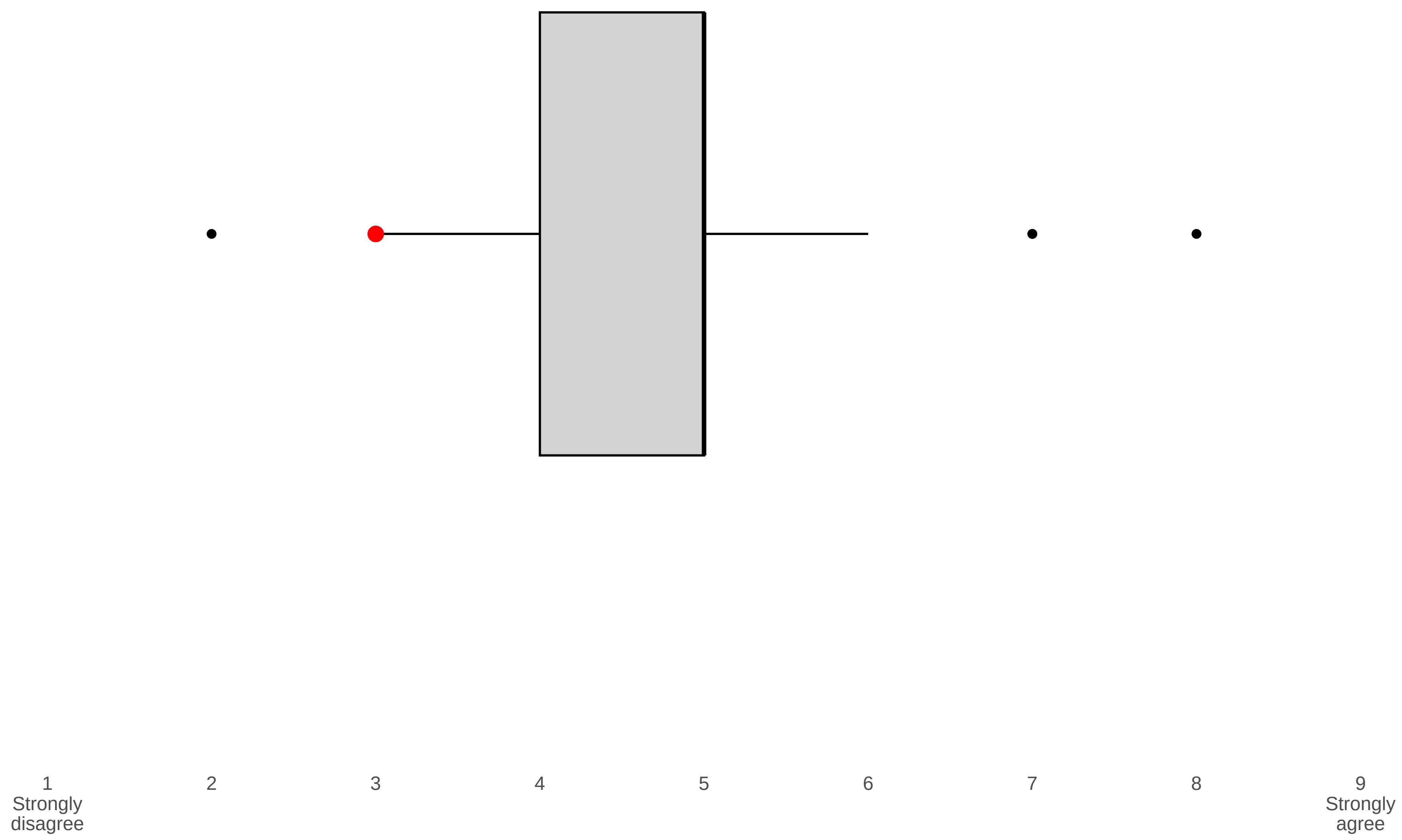

Reduced mini−mental state examination (MMSE) with a score of 20−24 is incompatible with driving.

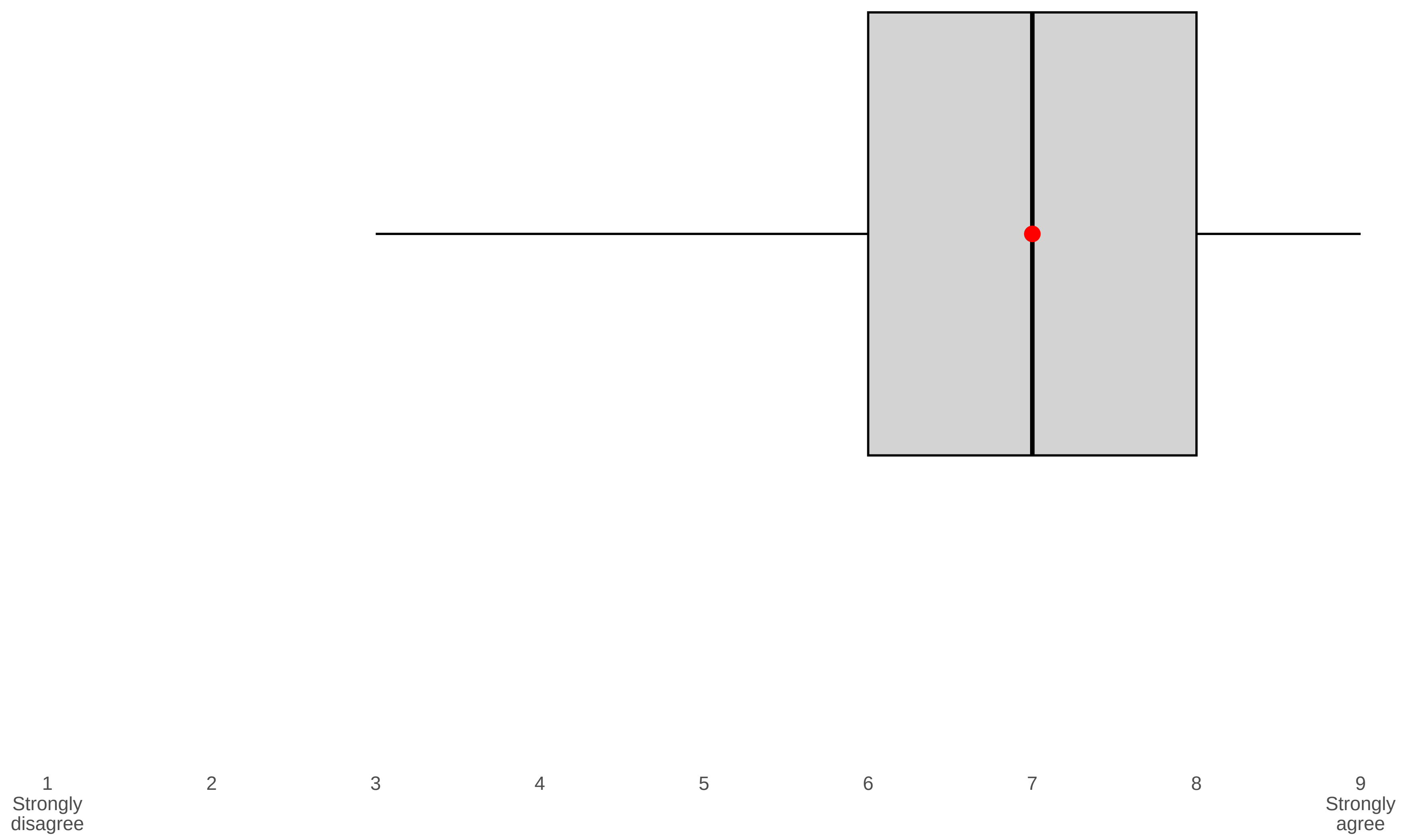

Reduced mini−mental state examination (MMSE) with a score of 10−20 is incompatible with driving.

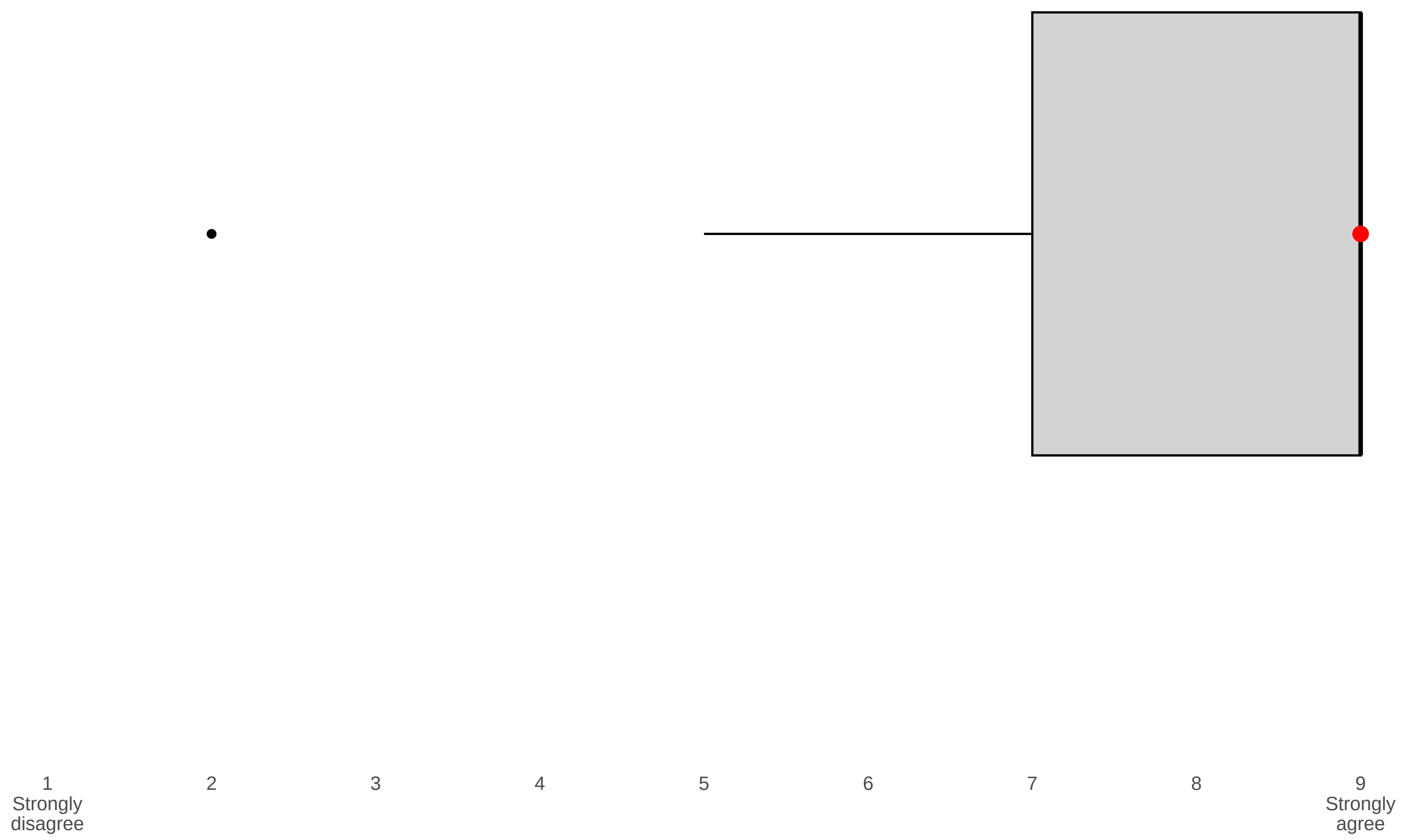

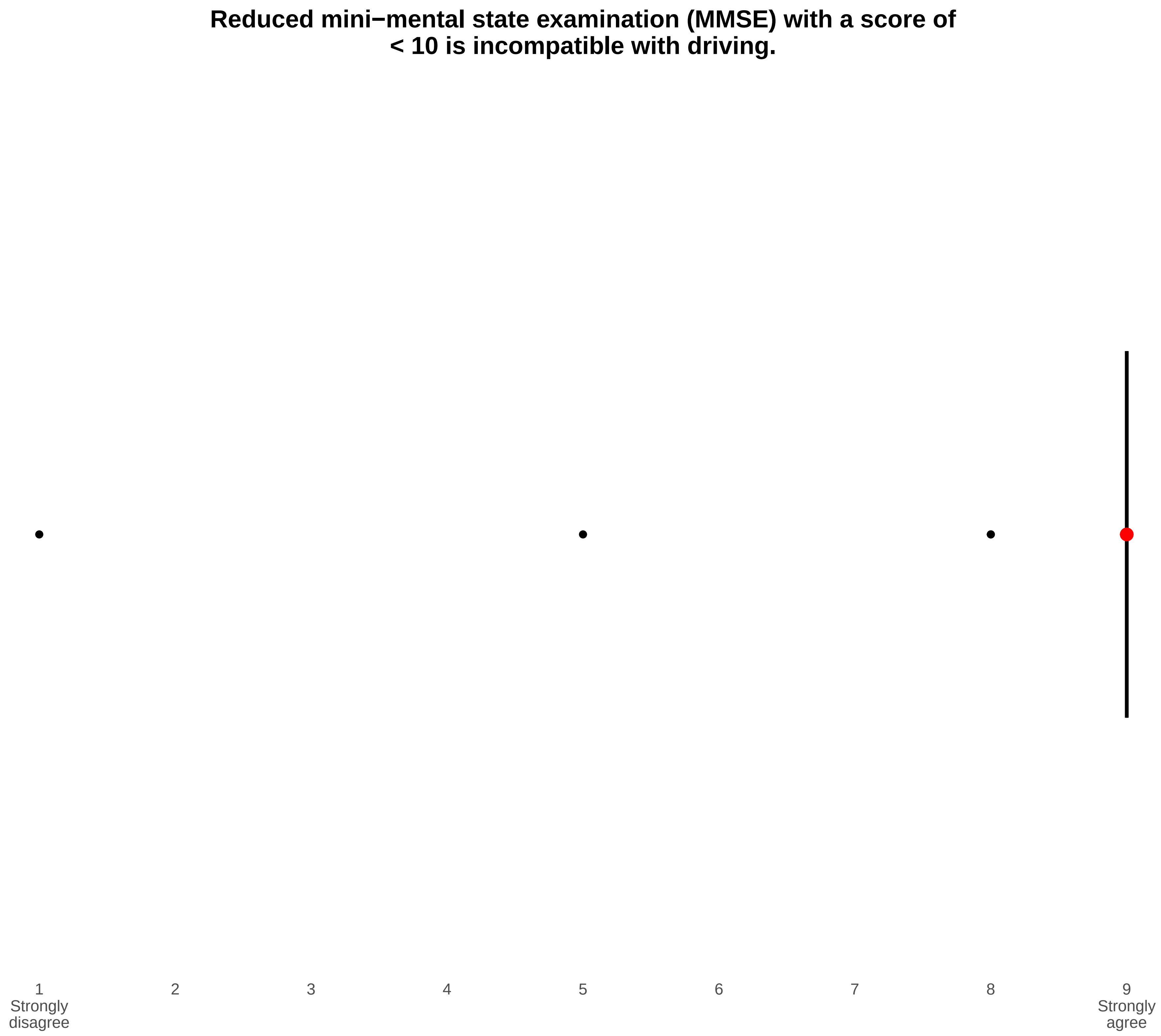
Reduced mini−mental state examination (MMSE) with a score of
< 10 is incompatible with driving.
1
Strongly
disagree
2
3
4
5
6
7
8
9
Strongly
agree

Glioblastoma tumour histology is incompatible with driving.

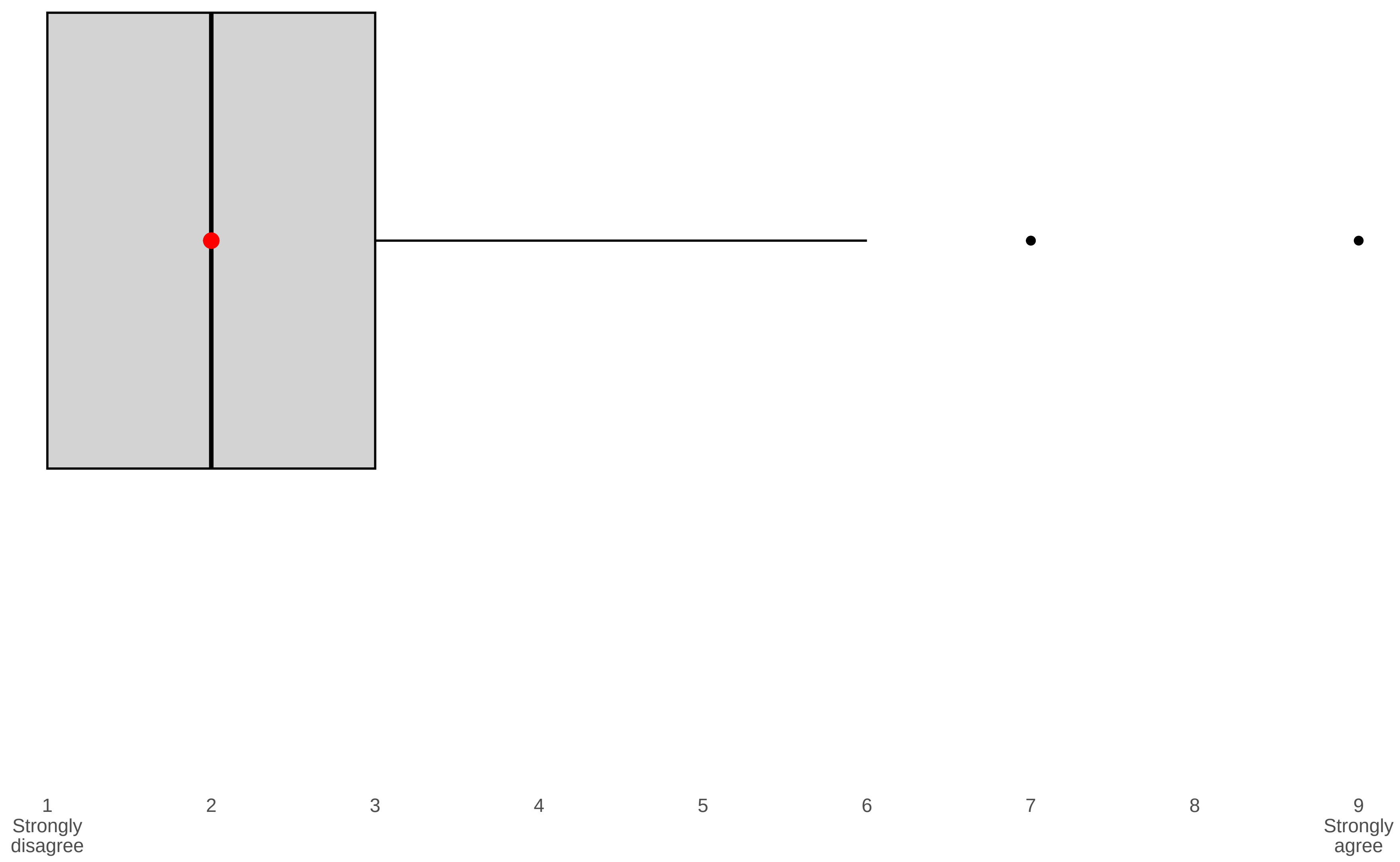

Metastatic carcinoma histology is incompatible with driving.

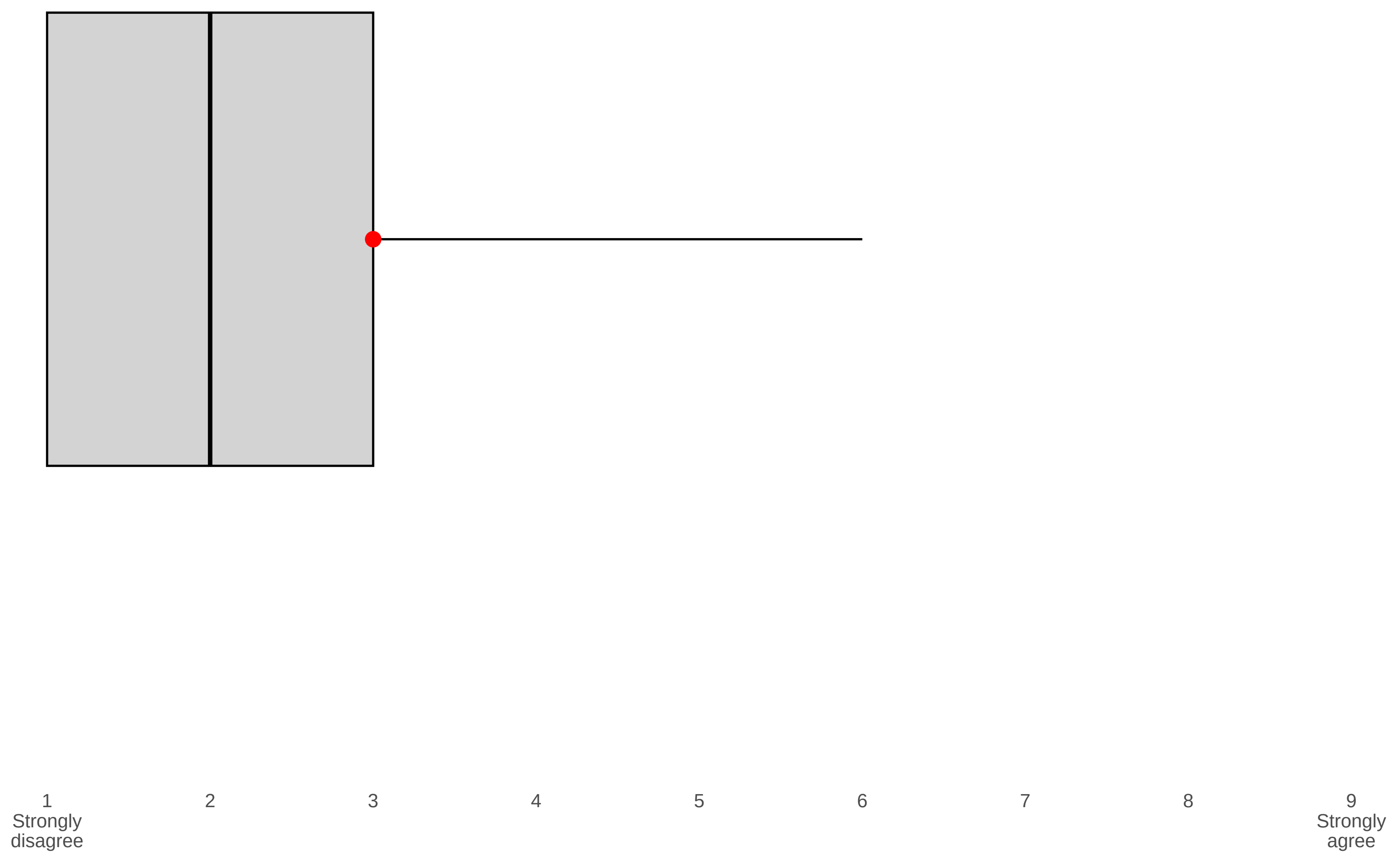

The unreliability of a patient is incompatible with driving.

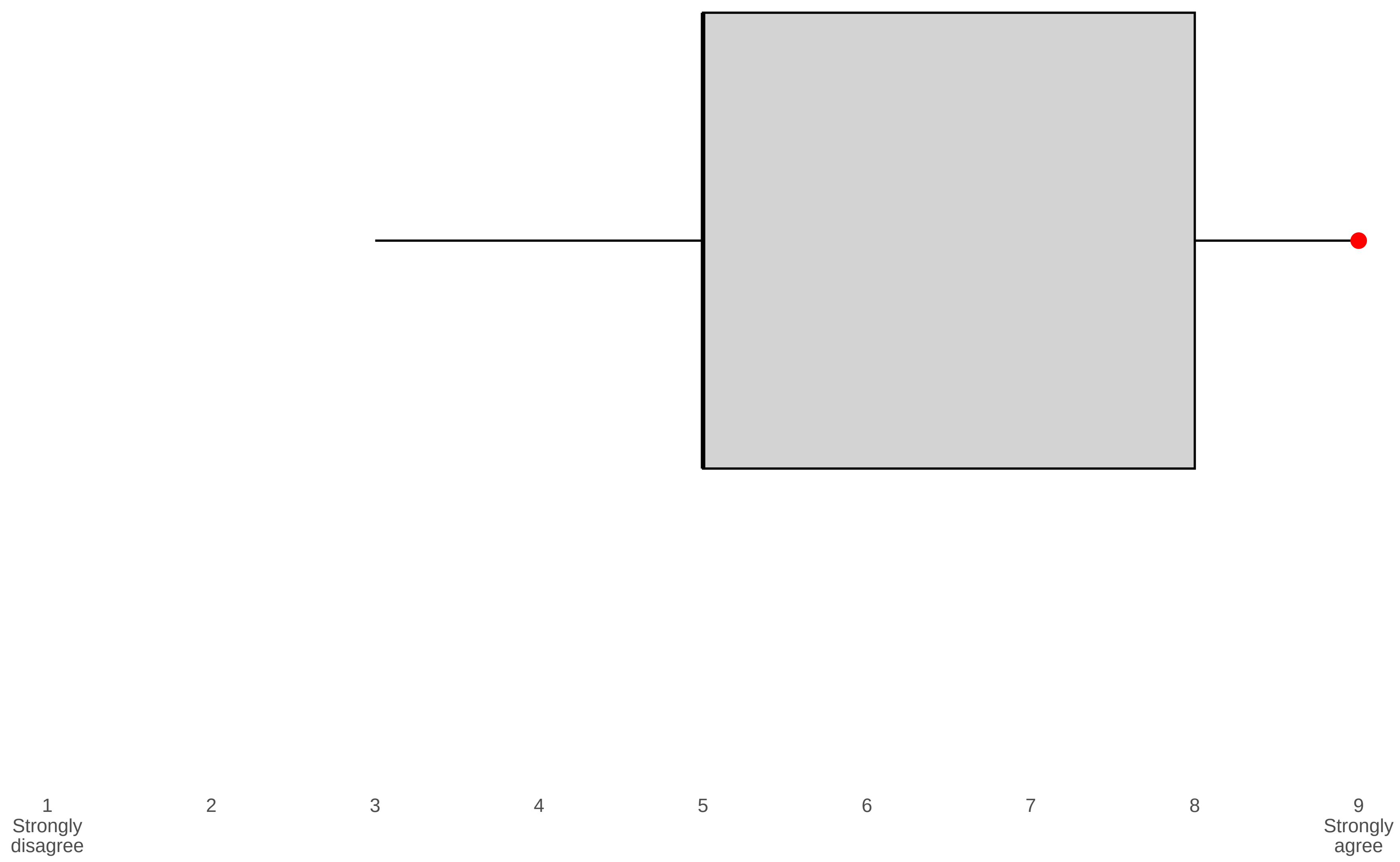

Questions That Achieved Consensus (Page 1 of 1)

Question	Consensus Type
For determining fitness to drive: A comprehensive neurological history is necessary.	high
For determining fitness to drive: A neurological examination is necessary.	high
For determining fitness to drive: A defined seizure free period is necessary.	high
The use of anti−epileptic medications is incompatible with driving.	low
The presence of ongoing seizures is incompatible with driving.	high
The presence of hemianopia is incompatible with driving.	high
Reduced mini−mental state examination (MMSE) with a score of 10−20 is incompatible with driving.	high
Reduced mini−mental state examination (MMSE) with a score of < 10 is incompatible with driving.	high
Glioblastoma tumour histology is incompatible with driving.	low
Metastatic carcinoma histology is incompatible with driving.	low

Questions That Did Not Achieve Consensus (Page 1 of 2)

Question	Consensus Type
For determining fitness to drive: Baseline (at the time of initial driving assessment) visual fields are necessary.	none
For determining fitness to drive: A formal visual assessment is necessary.	none
For determining fitness to drive: A baseline (at the time of initial driving assessment) neurological assessment with a focus on safe driving is necessary.	none
For determining fitness to drive: A stable repeat CT brain or MRI brain is necessary.	none
For determining fitness to drive: A driving assessment with a driving instructor / occupational therapist is necessary.	none
A defined period post−operatively if relevant is necessary.	none
The reduction or cessation of antiseizure medications should significantly influence driving restrictions for patients with brain tumors.	none
The status of active treatment or the time since the last treatment should significantly impact driving restrictions for patients with brain tumors.	none
I think it is important to continually reassess fitness to drive.	none
I believe the re−assessment should follow the same principles as the original assessment.	none
Patients should sign a document with the recommendation of their physician.	none
The current epilepsy guidelines for seizure−free period are adequate for patients with brain tumours / following intracranial surgery.	none
Each brain tumour subtype needs its own specific recommendation.	none
The presence of sensory neurological deficits is incompatible with driving.	none
The presence of motor neurological deficits is incompatible with driving.	none
The presence of cognitive neurological deficits is incompatible with driving.	none
The presence of an EEG with epilepsy−specific potential is incompatible with driving.	none
The presence of superior quadrantanopia is incompatible with driving.	none
The presence of inferior quadrantanopia is incompatible with driving.	none
Impaired executive function is incompatible with driving.	none

Questions That Did Not Achieve Consensus (Page 2 of 2)

Question	Consensus Type
Impaired visuospatial function is incompatible with driving.	none
Reduced speed of information processing is incompatible with driving.	none
Reduced mini−mental state examination (MMSE) with a score of 25−29 is incompatible with driving.	none
Reduced mini−mental state examination (MMSE) with a score of 20−24 is incompatible with driving.	none
The unreliability of a patient is incompatible with driving.	none
